# Neural mechanisms of attending to items in working memory

**DOI:** 10.1016/j.neubiorev.2019.03.017

**Published:** 2019-06

**Authors:** Sanjay G. Manohar, Nahid Zokaei, Sean J. Fallon, Tim P. Vogels, Masud Husain

**Affiliations:** a Nuffield Department of Clinical Neurosciences, University of Oxford, OX3 9DU, United Kingdom; b Department of Experimental Psychology, University of Oxford, United Kingdom; c Centre for Neural Circuits and Behaviour, University of Oxford, United Kingdom; d Oxford Centre for Human Brain Activity, University of Oxford, United Kingdom

**Keywords:** Working memory, Attention, Neural networks, Hebbian plasticity, Attractor network

## Abstract

Working memory, the ability to keep recently accessed information available for immediate manipulation, has been proposed to rely on two mechanisms that appear difficult to reconcile: self-sustained neural firing, or the opposite—activity-silent synaptic traces. Here we review and contrast models of these two mechanisms, and then show that both phenomena can co-exist within a unified system in which neurons hold information in both activity and synapses. Rapid plasticity in flexibly-coding neurons allows features to be bound together into objects, with an important emergent property being the focus of attention. One memory item is held by persistent activity in an attended or “focused” state, and is thus remembered better than other items. Other, previously attended items can remain in memory but in the background, encoded in activity-silent synaptic traces. This dual functional architecture provides a unified common mechanism accounting for a diversity of perplexing attention and memory effects that have been hitherto difficult to explain in a single theoretical framework.

## Highlights

• Evidence suggests both sustained activity and synaptic plasticity support working memory.
• Rapid Hebbian plasticity can support flexible attractor states analogous to a focus of attention.
• Plastic attractors can account for dynamic neural shifts in memory representations.

## 1 Introduction

Our capacity to hold and manipulate information over delays of a few seconds has long been thought to be subserved by the persistent firing of neurons during the delay (Funahashi, 2017; Fuster and Alexander, 1971). However, a number of recent studies have instead proposed “activity-silent” working memory, in which synaptic weights hold information during the delay, even in the absence of neuronal firing (Silvanto, 2017; Sreenivasan et al., 2014; Mongillo et al., 2008; Stokes, 2015). This dispute comes at a time when it is also becoming clear that working memory (WM) is not a homogeneous store. When we hold multiple items in WM, strong attentional effects are apparent. For example, people are faster and more accurate to recall the last item encoded, or the last item that was brought to mind (Chun et al., 2011; Oberauer, 2002; Souza and Oberauer, 2016; Zokaei et al., 2014a). First, we review how active and silent working memory have previously been modelled independently, and second, we build a simple neural model in which sustained firing and activity-silent working memory are reconciled as attended and unattended items within memory.

One item in memory, termed the ‘focus of attention’, appears to be in a privileged state. An item may enter the focus of attention when it is newly encountered, or if it becomes relevant for subsequent decisions or actions (Olivers et al., 2011). The identity of the focused item is decodable using functional MRI and is susceptible to TMS, unlike the unfocused items which are considered to be stored but in a non-privileged state (Lewis-Peacock et al., 2012; Sprague et al., 2016). In contrast, unfocused items are decoded better after their latent representation is re-activated (Rose et al., 2016; Wolff et al., 2017). These findings suggest that both active and inactive representations may coexist in WM, and items can move between these two states (LaRocque et al., 2014; Postle, 2016; Zokaei et al., 2014b). Computational neural models of both active (Compte et al., 2000; Zenke et al., 2015) and silent (Mi et al., 2017; Mongillo et al., 2008) WM have been separately postulated, but neither type of model on their own accounted for shifts of attention within WM. In Section 1 we review these models. In Section 2 we propose a model to account for both persistent activity and silent synaptic storage, that reproduces several neural and behavioral results regarding the focus of attention within memory, and makes new testable predictions. Section 3 discusses some open questions regarding models with this dual functional architecture.

### 1.1 Models of synaptic WM without sustained activity

Rapid synaptic plasticity at the millisecond scale has been used to explain how a pattern of inputs can be remembered (Fiebig and Lansner, 2017; Sandberg et al., 2003). In these synaptic models, simultaneously-activated neurons become more strongly connected. Whereas some models have utilized short-term facilitation (Mongillo et al., 2008), others have proposed Hebbian plasticity, which requires coincident firing of presynaptic and postsynaptic neurons (Fiebig and Lansner, 2017; Sandberg et al., 2003). Short term facilitation permits weakly-encoded activity patterns to spontaneously reactivate, allowing partial readout of those patterns (Trübutschek et al., 2017).

Plasticity has long been demonstrated in cortical neuron receptive fields (Edeline et al., 1993) and may arise through a variety of synaptic mechanisms (Zucker, 1989; Tsodyks and Markram, 1997; Fischer et al., 1998; Dittman et al., 2000; Jensen et al., 1996; Malsburg, 1981). In particular Hebbian learning rules allow associative mappings to be formed between neurons that are co-active, so that when a partial pattern is later presented, the original combination of active neurons can be re-activated, by associative recall. Such short-term Hebbian plasticity has been demonstrated in pyramidal neurons, is dependent on postsynaptic NMDA receptors, has a rapid onset after brief stimulation (e.g. after just 25 spikes over 500 ms) and may persist for up to 15 min (Malenka, 1991). Stronger stimulation may lead to facilitation over longer time scales, which may underlie associative episodic memory (Burgess and Hitch, 2005; Rizzuto and Kahana, 2001), or long term memory, which can provide the synaptic backdrop to support an active WM (Litwin-Kumar and Doiron, 2014, 2014; Zenke et al., 2015). Rapid plasticity in auto-associative networks can also account for serial recall of sequences of items (Fiebig and Lansner, 2017; Howard and Kahana, 2002) – including serial order effects such as primacy and recency (Farrell and Lewandowsky, 2002) – because new information may use up free space, or overwrite old information (Matthey et al., 2015; Sandberg et al., 2003).

One model of this kind uses associative plasticity, not between the co-occuring features themselves, but between the features and a separate ‘context vector’. Such *temporal context* models have been used to explain episodic retrieval (Howard and Kahana, 2002). In these models, input patterns co-occurring in time are bound by Hebbian plasticity to a temporal context — a vector which varies depending on the input itself.

In these synaptic models, the physiological meaning of a neuron’s firing depends upon its input and output connections. Plasticity in these models could therefore lead to neurons whose activity represents different things on different trials – a property that we characterize here as *flexible coding*. Such models may therefore generate novel testable predictions about neurophysiological data. However these models do not produce stable persistent-activity states in feature-selective neurons, which has long been considered a hallmark of WM (Funahashi, 2017).

### 1.2 Models involving sustained neural firing

In contrast, in sustained activity models, items are held in WM by virtue of delay-period activity (Compte et al., 2000; Funahashi, 2015; Funahashi et al., 1989), which relies on positive feedback to allow stimulus-induced activity to persist or resonate, leading to an “attractor” state. (Chumbley et al., 2008; Wimmer et al., 2014; Zipser et al., 1993). Although such active maintenance may also depend upon rapid changes in synaptic weights (Hansel and Mato, 2013; Pereira and Wang, 2015), the neurons generally retain their selectivity over time. These models do not generally allow memory recall from a silent inactive state.

Several non-plastic models have been proposed, in which features are bound by persistent activity in fixed conjunctive neurons. Fixed conjunctions may involve a spatial map (e.g. Schneegans and Bays, 2017a), neurons with mixed selectivity (Matthey et al., 2015; Schneegans and Bays, 2017b) or a “binding pool” (Bowman and Wyble, 2007; Swan and Wyble, 2014). In all these hard-wired models, information is stored only in the *activation* of neurons – not in their synapses. They must therefore overcome a combinatorial problem by employing lower resolutions (over low-level features) for the conjunctive neurons. These models predict that binding neurons should exhibit mixed selectivity, as observed in prefrontal cortex (Parthasarathy et al., 2017; Rigotti et al., 2013). The bottleneck also allows such models to predict interference errors, and may also account for some attentional effects on decodability (Schneegans and Bays, 2017a) but they cannot reinstate information that becomes fully undecodable from activity. With the exception of the binding pool which includes token- or pointer-like representations (Swan and Wyble, 2014), these models account for WM primarily as perceptual storage, in sensory brain areas. They do not explain how other brain areas read out or decode the stored information. Models involving spatial feature maps (Schneegans and Bays, 2017b) account also for the privileged role of spatial features, but they would require an analogous ‘map’ of temporal context to account for sequential same-location items.

An attractive common feature of several of these active-storage models is that the statistics of recall errors are accurately explained by interference, governed by the proximity structure of features within each dimension (Oberauer and Lin, 2017). Thus, if two items are nearby on a feature dimension e.g. space or time, they are more likely to be confused – as supported by behavioural data. In fact, this general result of Oberauer and Lin applies both to associative context models and the fixed conjunctive neuron models.

### 1.3 A new model of WM using a plastic attractor

The present work unites persistent activity attractors with silent synaptic storage. In our new class of memory model, both active and silent representations are essential to WM. We propose that persistent activation serves as the *focus of attention* that encodes recent activity patterns into synapses. Rapid plasticity in flexibly-coding neurons allows features to be bound together into objects, with an emergent property being that the last item is maintained actively. Recent, previously-attended items are preserved instead in synaptic traces. They are in a non-privileged state but, importantly, can be re-activated by partial information.

**Fig. 1:**
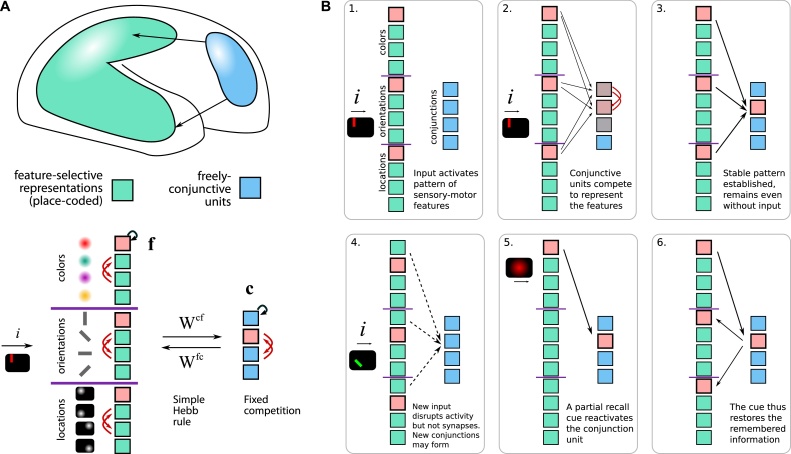
Conjunctive neurons form a plastic attractor to support attention and working memory. **A** Two populations of neurons are distinguished based on their inputs. Posterior neurons (green) encode sensory-motor features, whereas prefrontal neurons (blue) are “conjunctive”: i.e. they are able to rapidly increase or decrease their synaptic connectivity with patterns of feature neurons, using a Hebbian associative rule. We simulated 12 feature-selective neurons (**f**) and 4 freely-conjunctive neurons (**c**). An active combination of neurons (pink) causes strengthening of synapses in both directions, producing a stable attractor across brain areas. **c**=conjunctive cells, **f**=feature cells. W = synaptic weights, ***i***=sensory input. **B** Sequence of proposed neuronal events during attention, encoding and retrieval in working memory. **1.** Sensory input activates features. In this case a vertical red bar located at the top left of the display activates separate feature neurons tuned to orientation, color and location. **2.** Features excite conjunctive neurons, which compete. **3.** The winning conjunction drives sustained activity. **4.** New input to the system (in this case an oblique purple bar at bottom left) disrupts current firing activity, but synaptic weightings remain. **5.** Probe feature (in this case red colour) re-activates the original conjunctive unit that encoded the red vertical bar. **6.** Conjunctive unit re-activates original features, completing recall.

We propose that attention arises from the interaction between two distinct types of neural representation: fixed *feature* neurons, and *freely-conjunctive* neurons (Fig. 1A). Feature neurons may be sensory, motor or conceptual. They have fixed receptive fields or tuning curves – as observed in posterior cortical areas. In contrast, the freely-conjunctive neurons can rapidly change their connection weights with the feature cells, and therefore their activity does *not* represent a fixed feature or item in memory. Instead, through rapid plasticity on each trial, a conjunctive cell will come to encode a conjunction of simultaneously active features, by forming a transient reciprocal associative mapping to feature-selective neurons.

Persistent activity arises by mutual excitation between feature and conjunction neurons. The conjunction neurons form a limited-capacity store that can hold many kinds of information in one place. Thus, our model bridges the gap between neuron-level descriptions and the psychological notion of a *general-purpose register*, sometimes termed a “memory slot” (Cowan, 2010; Luck and Vogel, 1997), a concept which has not as yet been characterized at the level of single prefrontal neurons. Such registers are difficult to explain unless individual neurons can encode different types of WM content at different times. Our model permits this by allowing rapid synaptic changes so that conjunctive neurons can represent many kinds of information, depending on the recent context.

We suggest that two lines of evidence point to such conjunction neurons being located in prefrontal cortex (PFC): firstly, PFC is highly active in memory and manipulation (Eriksson et al., 2015; Postle et al., 2006), yet secondly, information is not always easy to decode (Christophel et al., 2012; Cogan et al., 2017; Kamiński et al., 2017). Although WM contents can undoubtedly be decoded from many PFC neurons, about 60% of prefrontal neurons appear to be nonselective, and even for those that are selective, they often show less than a 50% modulation of their firing rate by information in WM (Miller et al., 1996; Parthasarathy et al., 2017). This apparently-nonselective component of prefrontal activity could reflect transient and flexible coding by conjunctive units.

We first aim to provide a single common mechanism accounting for a diverse range of perplexing attention and memory effects. Second, we attempt to explain neurophysiological data where items in memory initially produce persistent activity, which then falls “silent” when attention shifts to new information (Konecky et al., 2017), and why sometimes “inverted” representations of unattended information may be decoded. Third, we aim to explain why many imaging studies conclude that attention and working memory are “distributed” processes involving both prefrontal and sensory brain areas (Christophel et al., 2017; Gayet et al., 2017, 2017; Xu, 2017) that also explain how WM enables us to encode and execute task rules. In our simulations, we chose to examine the extreme situation where conjunctive neurons are fully nonselective for features. This limiting scenario is of course implausible, since no single prefrontal neuron could receive input from every feature neuron. However we argue that it is a highly illustrative paradigmatic case. In reality prefrontal neurons will necessarily have some degree of selectivity, but here we focus only on characterizing the novel concept of how rapid plasticity can give rise to flexible coding, and therefore we model *purely* conjunctive neurons as distinct from feature-selective neurons.

## 2 Simulation of a generic feature binding model

### 2.1 Operation of the model

When a stimulus is perceived (Fig. 1B; Movie S1), conjunctive neurons compete through lateral inhibition to become active in response to the combination of active features. In the example shown in Fig. 1 the conjunction units learn rapidly to encode combinations of color, orientation and location (Fig. 1B.2). During encoding into WM, the winning conjunctive unit sustains the activity of all co-active feature neurons through mutual excitation. This strengthens synapses in both directions through rapid Hebbian plasticity, further stabilizing the active pattern. Once a conjunctive unit succeeds in reciprocally activating a set of feature units, we say that *attention is focused* on the activated features, binding the features of a compound stimulus into a perceptual object.

The reciprocal feature-to-conjunctive synapses keep the novel combination of features persistently active, even when the stimulus is no longer present (Fig. 1B.3).

When a new stimulus arrives, a new pattern of sensory input destabilizes internal activity, shifting activity away from the attractor carved by the first object. A new conjunction may win and form another attractor state by plasticity, which in our model amounts to shifting the focus of attention to the newly activated feature pattern. Crucially, however, synapses between the previous object’s constituent features and one particular conjunctive unit remain strengthened even after those neurons fall silent (Fig. 1B.4). Thus, presenting any one feature of a previously attended object (e.g. color, as shown in Fig. 1) will act as a memory probe, re-activating the corresponding conjunction neuron (Fig. 1B.5), and therefore also the other features that were associated with it (Fig. 1B.6). The object’s features are therefore recalled by auto-associative pattern completion, which brings them back into an attended, foreground state. Separate objects must always be encoded sequentially, which we suggest is plausible in light of the empirically observed attentional bottleneck in feature binding (Reynolds and Desimone, 1999).

**Fig. 2:**
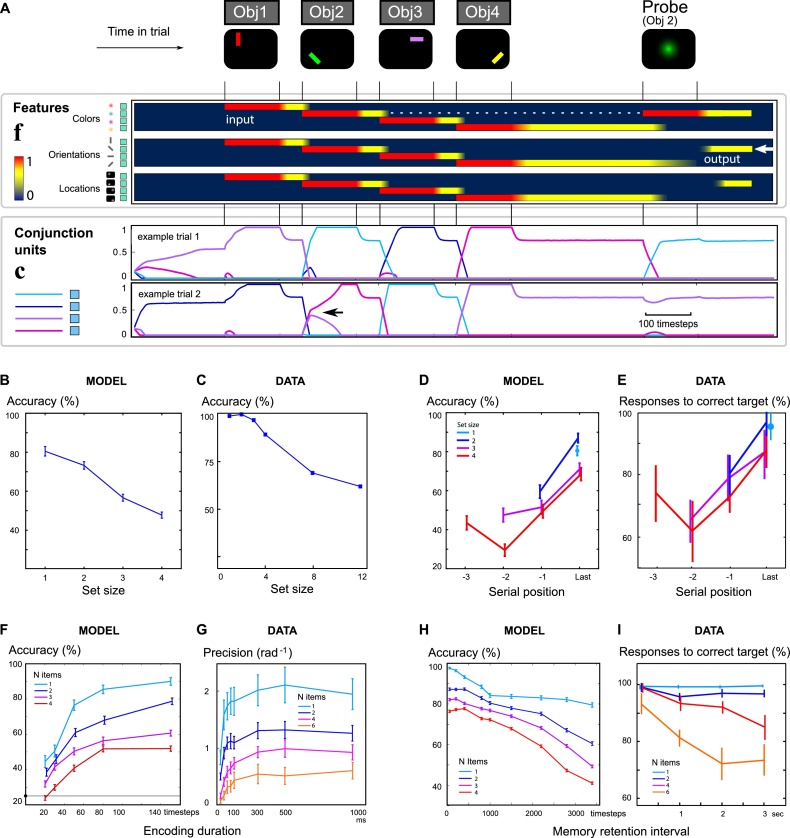
Predicting visuospatial WM capacity, encoding and decay. **A** To simulate WM performance, four objects are presented sequentially, by activating feature neurons (**f**, activity depicted as a heatmap from dark blue to red) indicating the color, orientation and location of each item. Conjunctive units (**c**) are shown below as four differently-colored traces. Conjunctive units compete to become active for each object. One conjunctive unit wins for each object, driving activity that persists even after input is removed (yellow parts of heatmap). At the time of the probe, a single feature is stimulated, triggering pattern completion. Recall is accurate if the orientation of the corresponding item is re-activated. Two example trials are shown; note that different patterns of conjunctive units are activated on different trials even for the same stimuli, depending on trial history. Example 1: good encoding. Example 2: weak encoding of the second item. Two conjunctive neurons with similar recent preferences compete to encode object 2 (arrowhead). When it is probed, item 4 is reported instead. **B & C** When more items are encoded in the model, recall accuracy is reduced, as observed in data (adapted from Luck and Vogel, 1997). **D & E** The last item encoded in the model is recalled better than others, as it remains active in the focus of attention during the delay period, matching observed serial order curves. Figure adapted from (Gorgoraptis et al., 2011) indicates the probability of reporting the target item as calculated by fitting the distribution of responses in a similar task. **F&G** Shorter encoding durations reduce modelled recall accuracy. Data from a similar task (adapted from Bays et al., 2011b) where adding items reduced both initial encoding rate and asymptote. The model qualitatively reproduces the interaction observed in human performance. **H & I** The model predicts faster memory decay when more items are stored. This matches the empirical interaction between memory-set size and delay. Data adapted from (Pertzov et al., 2016) shows the modelled probability of reporting the target. Note that at very short delays, model recall was more accurate than in human data.

To demonstrate the power of the model, we simulated a common visuospatial WM task (Fig. 2A) in which participants remember the orientations of a set of colored bars (e.g. Gorgoraptis et al., 2011; Pertzov et al., 2016). Neurons were modelled as firing-rate units obeying a Hebbian plasticity rule (see Methods). Memory items were composed of combinations of features, and up to four unique items were presented sequentially to the feature units. After a delay, we probed one of the items by activating its color-feature alone, and recording whether its orientation was subsequently re-activated. Remarkably, just four color, orientation, location and conjunctive neurons each are needed to explain a wide range of behavioral and neurophysiological data, which no models have yet captured (Table S1).

Crucially both the activation and learning equations were implemented continuously over a block of trials, with blank input in between trials, so that encoding, recall and interference from the previous trial all arose naturally from the way stimuli were presented. We tuned the model to perform at levels comparable to humans at this task (see Methods).

For clarity, here we elected to keep the model’s operation almost identical for all the simulations, even though the experimental data we match come from a variety of tasks and measures. Although it is possible to adjust the numbers of features, synaptic and learning parameters and timings to reproduce each individual experiment, this permits many degrees of freedom. Thus we believe that showing that a single generic model can qualitatively reproduce all the effects makes clear the capabilities and limitations of the basic model. Moreover, we wished to emphasise that features within the model could also map to non-visuospatial attributes – such as motor or conceptual representations.

First we confirm the network can qualitatively produce standard working memory effects; second, we demonstrate the novel features of the model regarding the focus of attention; third, we show that the model accounts for neural data from multi-item WM; and finally, we make some novel predictions and show that model could be extended to implement task rules.

### 2.2 Capacity limits and serial order in WM

A key feature of WM is its limited capacity. The more items held in memory, the less accurately they are remembered (Luck and Vogel, 1997; Bays and Husain, 2008). Simulated recall accuracy (Fig. 2B) matched the set-size effect from classical visuospatial WM experiments (Fig. 2C). This is because each additional stimulus competes for conjunctive neurons, and may corrupt or overwrite synaptic traces of previously-seen objects. Whether a previous item is overwritten is determined by how well the currently-active features match the existing synaptic weights, which are themselves continuously subject to Hebbian rules. Therefore in our model, capacity is limited by interference between items in memory, similar to several previous psychological models (Howard and Kahana, 2002), in line with convergent evidence from multiple WM domains (Almeida et al., 2015; Farrell et al., 2016; Oberauer and Lewandowsky, 2014). Note that with our canonical example parameters, accuracy is lower than the illustrated data because the model chooses between four options rather than two, but varying the model parameters can make it arbitrarily more accurate (Fig. S8,S9). Moreover the capacity limit is not simply determined by the number of conjunctive neurons, and can be adjusted by tuning the level of inhibition if more conjunctive neurons are used (Fig. S14).

Importantly the model predicts the counterintuitive finding that storing extra features on different dimensions within a single object either occurs automatically (Allen et al., 2006) or else may incur a smaller cost than a separately-encoded feature (Luck and Vogel, 1997) – although other studies have demonstrated that extra features do impose costs (Oberauer and Eichenberger, 2013). Our model predicts that primacy effects may be stronger when adding an irrelevant but distinguishing feature to each object (Fig. S7). Controversially, some studies indicate that objects form fundamental units (Hardman and Cowan, 2015; Luria and Vogel, 2011), whereas others suggest show that the features of an object can be forgotten independently (Bays et al., 2011a; Fougnie and Alvarez, 2011; Wang et al., 2017). Our model predicts a mixture of feature-based and object-based forgetting (Fig. S17).

### 2.3 Serial order effects

When we remember a sequence of objects, we recall the first and last objects better (primacy and recency). Our model can reproduce both of these effects. Simulated performance (Fig. 2D) matched the serial position curve obtained in WM experiments (Fig. 2E). The simulation suggests that neutrally, primacy benefits arise because the first object in a trial does not need to compete with ongoing persistent activity from a previous item (Fig. 1B4). In our model this relies on the fact that, at the start of each trial, feature units are inhibited but previous synaptic weights are not erased – though there is no explicit signal to forget items from the previous trial. Recency benefits arose for two reasons. First, the finally-encoded item did not incur retroactive interference from subsequent items, whereas previous items are corrupted by interference when subsequent items are encoded. Second, the final item remains in an active state rather than a silent state during the delay. Note that our plasticity rule has no explicit temporal decay. Because capacity limits are generated through interference, we only require that the plasticity lasts longer than the memory delay (Fig. S11). Serial position effects are strongly disrupted when items share features (Fig. S16).

### 2.4 Encoding and maintenance

The time-course of encoding was interrogated by presenting items for brief durations, and demonstrated exponential saturation with an asymptote dependent on the number of items encoded. In a similar empirical study (Bays et al., 2011b), memory precision (1/standard deviation of response angular error) followed a similar pattern. In that study, the probability of choosing the target was not calculated, but their reported precision appears to correspond well to our model’s probability of reporting the correct target orientation (Fig. 2F&G). Accuracy and precision are not guaranteed to be equivalent measures however.

Simulations demonstrated that memory deteriorates faster when increasing numbers of items are remembered (Fig. 2H&I), as shown in a recent study (Pertzov et al., 2016). This arises because a greater proportion of items are held in an unattended state. Unattended items are more vulnerable to interference, because their synapses are gradually weakened over time. This occurs not because of any specific decay rule, but rather because the plasticity rule operates continuously to alter all synaptic weights, and this ‘erodes’ the representations that are not currently active, such that all non-attended features become more homogeneously connected to the non-attended conjunctive neurons (see Supplementary Video). Memory items therefore interfere with each other during the delay. Our model also makes the strong prediction that an item stored in an attended state (e.g. the final item in a sequence) is more robust to decay over time. For very short delays, the last item was recalled even better (akin to an “iconic” effect, Fig. S13), an effect that was not seen in human data.

### 2.5 Shifting the focus of attention

**Fig. 3:**
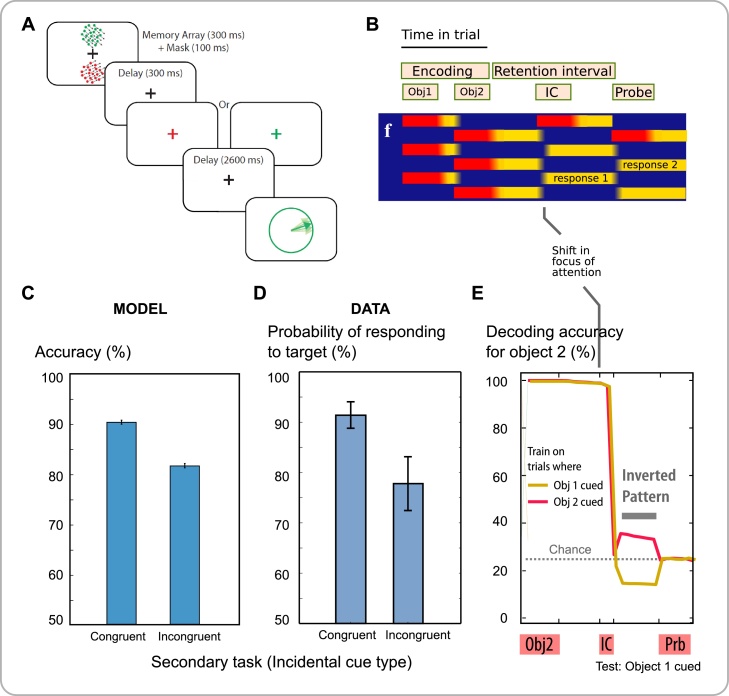
Shifting the focus of attention in WM. **A** Experiment (Zokaei et al., 2014a) where participants remembered two items, each comprising three features: color, location and orientation. During the retention interval, a color was shown, and as a secondary task, the location of the corresponding object had to be recalled. At the end of the delay, a color was shown which could indicate the same (“congruent”) or different (“incongruent”) object than the one tested during the delay. Participants then reported the orientation of the corresponding object. Reproduced under the terms of the Creative Commons Attribution 3.0 Unported (CC BY 3.0) license (https://creativecommons.org/licenses /by/3.0) from Fig. 1A of Zokaei et al. (2014a), The Journal of Neuroscience. January 1, 2014. 34(1);158–162. **B** Similar events were simulated, with an incidental cue (IC) during the delay. If the first object was cued, then persistent delay activity shifted to the cued item. **C&D** The model predicts that the item in the focus of attention before recall is reported more accurately, matching data. Probability of target from mixture model fitted to data of Zokaei et al. 2014. **E** Decoding direction of object 2 from feature-selective units during the delay, on trials where the first item was cued (IC). Decodability is low but still above chance after the cue, with below-chance performance of a cross-decoder trained on trials where the second item was cued (full analysis Fig. S12).

An important advance over other models, is the ability of our model to re-activate a previous item by bringing it into the focus of attention. The logic here is that sensory input can guide attention by pattern-completion. In behavioral experiments, an “incidental” task inserted into the memory delay can shift attention to one of the items in memory (Fig. 3A) (Zokaei et al., 2014a) bringing it into the foreground. We simulated “retro-cueing” one of the items during the memory delay by presenting one of its features for a brief period, which brought that item back into the focus of attention (Fig. 3B). The external cue could thus re-activate a memory item which was previously encoded silently. Note that this simulation illustrates how feature-selective units can exhibit task-dependent modulation because they also receive non-sensory input through rapidly-plastic synapses from the conjunctive units.

Recall of the incidentally-cued item improved, compared to the uncued item (Fig. 3C), matching experimental data (Fig. 3D). This attentional shifting also explains how cues that indicate which item will be probed (predictive retro-cues, Rose et al., 2016) improve performance.

### 2.6 Recall

After the probe feature was activated, it took a number of time steps for the conjunction and response feature units to become active. We measured this time to obtain reaction time predictions, which varied inversely with accuracy similar to empirical data (Fig. S1).

The process of recall may also be susceptible to interference, because it effectively uses pattern completion to re-activate the other features of the corresponding object. In particular, the memory probe itself can interfere with recall, for example if it contains a feature on the dimension that needs to be reported (Fig. S2), in line with empirical probe-interference effects (Souza et al., 2016). Items in the focus of attention are protected from probe interference, presumably because they do not need to be brought back from an inactive state (Makovski et al., 2008; Wang et al., 2018; Tabi et al. 2019) Interference of another kind arises when recalling items as a whole series: often the preceding or following item is reported instead (Smyth, 1996; Solway et al., 2012). Although our simulations probe a single item at a time, they still demonstrate such “transposition errors”, where consecutively presented objects are confused (Fig. S3).

### 2.7 Neural encoding of items in WM

**Fig. 4:**
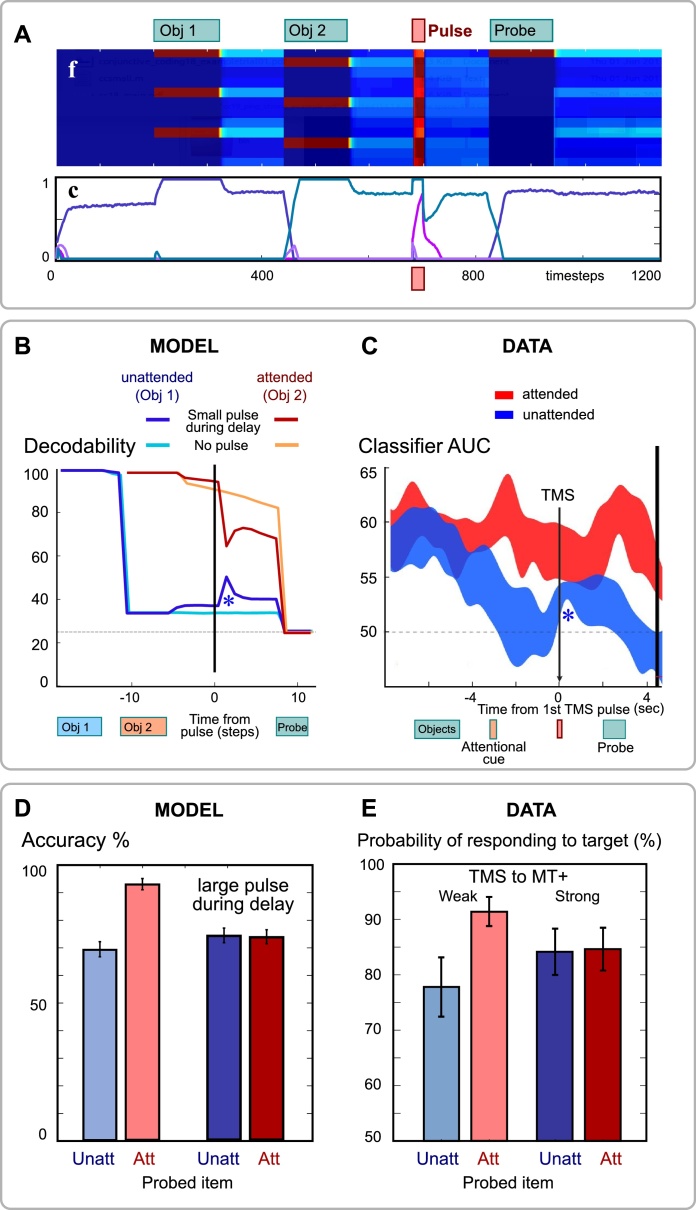
Introducing a pulse of excitation during the delay period. **A** After presenting two items, during the delay all feature neurons **f** received an excitatory input pulse **i**=+1, consequently activating conjunction neurons. **B&C** We tried decoding the identity of each of the two stimuli from feature neuron activity. Although the first object was not decodable without the pulse, it became transiently distinguishable (*) after the pulse. This matches the observed increase in decodability after TMS (Rose et al., 2016). **D&E** Stronger pulses altered model performance, abolishing the benefit for the second item, which was in the focus of attention. The pulse disrupted persistent activity, re-instating competition between conjunctive neurons. This results in randomly re-selecting which of the stable states of the plastic attractor is active. The prediction matches observed effects of TMS targeting motion-selective cortex (probability of selecting the target in mixture model fitted to data from (Zokaei et al., 2014a).

Three major predictions emerge about neural decoding. First, an emergent property of our framework is that sustained activity represents a single item held in memory (Funahashi, 2017), but not multiple items (Lara and Wallis, 2014). We used a linear decoder to extract information about one feature of one of the items in WM, after items had been encoded. The predictions of the model for decodability from feature-selective neurons (Fig. S4) are in keeping with human and nonhuman physiological data demonstrating that only the attended WM item is decodable using standard techniques (Konecky et al., 2017; Lewis-Peacock et al., 2012; Sprague et al., 2016). Second, evoking neural activity by stimulation can restore decodability from EEG signals (Rose et al., 2016; Wolff et al., 2017). We simulated transcranial magnetic stimulation (TMS) by an indiscriminate pulse of activation to feature neurons (Fig. 4A), and decoded one feature dimension from feature-selective units (Fig. 4B). If the model’s color and orientation feature dimensions are considered as mapping to spatial location and stimulus category respectively, then the simulation matches the effects of TMS on decoding (Fig. 4C) (Rose et al., 2016), or if they are instead mapped to spatial location and orientation, then the model’s results reproduces the effects of a high-energy visual pulse (Wolff et al., 2017). Simulating a stronger pulse of stimulation disrupted attention, but not synapses. This worsened recall of the attended item, yet contrarily improved unattended items (Fig. 4D&E), precisely as demonstrated empirically (Zokaei et al., 2014a).

We were initially surprised to note that even when the second item is in the focus of attention, during the delay period, decoding for the first item is still above chance. We therefore employed ‘cross-decoding’ to examine whether an item is encoded in the same way when it is attended vs unattended (Fig. S12). The accuracy with which a classifier could decode an item from the activity of feature neurons fell considerably *below chance* when training on attended and testing on unattended representations, and vice versa. Thus, unattended items were encoded in an ‘inverse’ pattern to the attended items. Why should this be? In our model, feature neurons of items not in the focus of attention are inhibited by the mutual competition in that layer. This led to a non-specific inhibition of the unattended three features in each dimension. Moreover, conjunctive neurons for unfocused objects are also inhibited by competition, leading to selective inhibition of neural pattern corresponding to the unattended item. Remarkably, several studies in recent months have suggested this “representational inversion” phenomenon can be observed in human imaging data (van Loon et al., 2018; Rademaker et al., 2018; Yu and Postle, 2018).

**Fig. 5:**
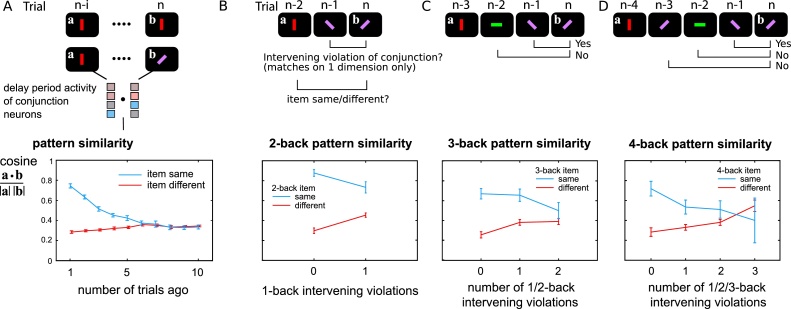
Conjunctive unit representations are stable over short timescales. Conjunctive units change their selectivity over short periods. If selectivity were stable, neural patterns should be similar when the stimulus is the same. We compared similarity of the pattern of an earlier trial, to trial *n,* during the delay periods of a series of 1-item trials. **A**) The similarity of the conjunctive neurons’ delay activity pattern is calculated for trials where the stimuli were identical (blue line) or different (red line). Patterns were more similar when stimuli were the same, compared to when stimuli were different, indicating “classical encoding” at least for nearby trials. This classical behavior decreased with the temporal distance between trials. Since we modelled the extreme case where neurons are *purely* conjunctive, with no feature selectivity, consistency of pattern is completely abolished after about 6 trials. **B-D**) The model predicts that interference reduces pattern similarity over time by overwriting the synaptic weights. If the objects in intervening trials share one feature with the *n*th trial object, but mismatch on the other feature dimension, then we say the conjunction between the two feature dimensions is “violated”. **B**) When the intervening trial contained a violation, the patterns on the *n*-2 and *n*th trials reflected the stimuli much more weakly, indicating interference or overwriting of the original conjunction. **C** and **D**) Trials 3-back and 4-back were similarly examined, this time asking how many intervening conjunction violations occurred. The more overwriting that occurred between the *n*-3 and *n*th trials, the less classical encoding could be observed.

Third, the model predicts that decoding from prefrontal cortex is unreliable (Lee and Baker, 2016). This is because the concept of a receptive field breaks down for conjunctive neurons. The same activity can have *different meanings* on different trials, dependent on residual synaptic weights from previous trials. Such neurons should show much stronger representations over short timescales. We predict this will manifest behaviorally, with better recall for a feature combination present on the previous trial (Fig. S5), because the same conjunction unit will be re-used. Moreover, neural activity patterns in conjunction neurons predict stimuli strongly if we consider data only from *contiguous* pairs of trials, compared to data from temporally-separated trials (Fig. 5A), and the pattern similarity should be even lower when intervening stimuli involve a recombination of the features (Fig. 5B–D). This confirms that each conjunctive neuron’s activity represents different things, as its synaptic weights change. Such a system can flexibly encode a broad variety of novel information rapidly, without incurring the combinatorial explosion that haunts previous fixed-selectivity models (Matthey et al., 2015; Postle et al., 2006).

### 2.8 Simulation of task sets

The same system can also implement stimulus-response rules, if some feature neurons represent motor plans. In this case, we encode a *task rule* by attending to a stimulus and a motor plan together. For example, if a left-hand movement plan is activated while a red color-feature is simultaneously activated, they will be encoded together into working memory. The conjunction of sensory features with a motor plan creates a task-set mapping (Duncan et al., 2012). Later, that stimulus can also re-activate the corresponding motor plan by pattern-completion, triggering the movement – so that the stimulus generates a response. Task sets can therefore be rapidly formed by sequentially attending to stimulus-response pairs (Curtis and D’Esposito, 2003), and deciding on an action is simply the motor analogue of WM recall.

To simulate stimulus-response mapping, we presented the task rules sequentially, each consisting of a pairing between one color and one response (Fig. S6A). Then on each subsequent trial, a single color from the set was shown, and the response was recorded. The model reproduces Hick’s law, in which response times are longer in situations when more response options are possible in the current task set (Fig. S6B) (Proctor and Schneider, 2017). It also produces faster reaction times when the response is repeated from the previous trial (Fig. S6C), in line with experimental evidence (Schvaneveldt and Chase, 1969).

In this situation, the role of prefrontal conjunctions can be viewed as *controlling* representations in posterior cortex, i.e. routing information from perceptual to motor representation as governed by task sets held in working memory, a role classically assigned to executive/supervisory attention. Critically the model predicts that, because the task rules are held in WM across many trials rather than being repeatedly overwritten, the current stimulus and response (i.e. the active task rule) are consistently decodable from conjunctive neurons, until the rules change (Fig. S6D). This contrasts with WM storage, where frequent overwriting leads to poor decoding, and may explain why task rules have generally been easier to decode from PFC (Reverberi et al., 2012; Sakai, 2008).

### 2.9 Simpler models

To investigate the necessity of various components of our model, we compared the full model with three variants with simpler assumptions. First, we examined a model that used non-Hebbian short-term facilitation at synapses between the feature and conjunctive neurons. With the small number of fully-connected neurons in our model, this is unable to generate stable persistent activity because facilitation is not synapse-selective (Fig. S10A). Second, we removed plasticity from just the conjunctive-to-feature neurons. This network was able to produce stable persistent activity, but was unable to re-activate appropriate features during recall, because the reciprocal synapses back to the feature neurons did not develop appropriate selectivity (Fig. S10B). Third, we examined a model without conjunctive neurons, but with Hebbian plasticity directly between feature neurons. This model was able to produce sustained activity, and could shift attention between items in memory. It accounts for set size and some aspects of the serial position curves, but did not produce interference between memory items during the delay (Fig. S10C). Moreover, without conjunctive neurons, we are unable to predict prefrontal activation during WM tasks, or the possibility of activity without apparent selectivity. Conjunctive neurons also potentially allow for extending the model to support gating of distractors and internally-driven shifts of attention, without invoking extensive prewired connectivity between feature-selective neurons and prefrontal cortex or thalamus. For these reasons, we conclude that a combination of Hebbian plasticity and flexibly conjunctive neurons are critical components for our particular model.

## 3 Discussion

The model of freely-conjunctive neurons presented here accounts for both sustained firing and activity-silent synaptic traces in WM (Silvanto, 2017; Stokes, 2015), and consequently makes a range of testable behavioral and neural predictions (Table S1). This neuronal framework provides a parsimonious mechanism for feature binding, general-purpose memory ‘slots’, and task sets. The model reproduces classical WM effects of capacity, serial order, encoding rate, temporal decay (Fig. 2), reaction times, and transposition errors (Figs. S1&3), as well as the ability to switch attention between items within memory – a phenomenon that evades most current models (Fig. 3). At a neural level, it explains why it is difficult to decode memory contents from prefrontal activity, why only the item in the focus of attention can be decoded elsewhere (Fig. S4). Further it explains why decodability can be restored by re-focusing an unattended item, or after a perturbation such as transcranial magnetic stimulation (TMS) or bottom-up input (Rose et al., 2016; Wolff et al., 2017), which presumably re-activate the conjunctive neurons and thus an object’s features through synaptic traces (Fig. 4). The model also makes strong novel predictions about probe interference, trial-to-trial effects (Figs. S2&5), and disruption of neural pattern similarity by intervening stimuli (Fig. 5).

### 3.1 Flexible neural codes

One strength of our model is that it allows pattern completion using flexible attractors, potentially providing a mechanism for mapping information in WM to appropriate responses, via changes in the focus of attention.

To support flexible attractor states, we postulated two distinct modes of neural representation (Fig. 1). First, feature-selective neurons are traditional, place-coded (“labelled-line”) units. They are selective because they have some fixed, non-plastic inputs (or in the case of motor units, fixed outputs). But if plasticity modifies both the input and output synapses of a neuron, the meaning or interpretation of a neuron’s firing will also change. This is simply because neurons *code* information only in virtue of their inputs and outputs. Plasticity therefore begets a new category of flexibly-coding neurons, where the information signaled by firing is protean and dependent on the history on each trial. Decoding the fine-grained identity of stimuli from prefrontal cortex is unreliable compared to posterior sensorimotor regions (Cogan et al., 2017; Lee and Baker, 2016), because the idea of a receptive field breaks down. Standard decoding methods assume trial-to-trial stability of activation patterns to represent a given feature, and so do not measure the sequential effects we predict.

This flexible coding scheme is crucial for our model to generate two phenomena. First, it permits sustained activity that is guided dynamically by task sets or objects in memory, which we postulate corresponds to attentional interactions between frontal and temporo-parietal regions. Second, because individual neurons can encode different things at different times, information must *compete* to be encoded by any conjunctive neuron – thus leading to a capacity limit for general-purpose information storage, observed in both WM and attention. This may help resolve a long-standing theoretical debate on whether working memory consists of pointers, or activated long-term memory (Norris, 2017): conjunctive neurons act as pointers that activate long-term memories. If the inputs to feature-selective neurons are governed by long-term memory, then their activity may convey abstract conceptual, configural or statistical information about the world, which could then also be flexibly controlled by conjunctive neurons. For example, it would be possible to store arbitrary associations–for example, “if you see a platypus, touch your nose with your left hand”, by holding the neurons active during the encoding of the rule that are selective for platypuses, moving the left hand, and targeting the nose.

The conjunctive neurons in our model mathematically resemble temporal contexts (Howard and Kahana, 2002), but those models have focused on behavioral effects in list recall. They do not explicitly keep one item in a privileged state, and make few direct neural predictions. Unlike the temporal context model, our model does not account for working and episodic memory in a single framework.

### 3.2 Relaxing the model’s assumptions

In this study we deliberately chose to study the simplest possible model that could support conjunctive neurons. The very small number of neurons, and their simple learning and dynamics, makes it much easier to see how they interact to generate the novel predictions. Moreover it is much more transparent where the model can or cannot match existing data. Naturally there are many directions in which the model needs to be extended, to fully reproduce the phenomena observed in real neurons. A number of its assumptions can plausibly be relaxed.

### 3.3 Pure flexible and stable representations

For simplicity we have treated conjunctive neurons as “pure”: i.e. that they are homogeneous and domain-general, resulting in inability to decode information across many trials. This architecture parallels the psychological notion of a memory slot. However it is certainly implausible because all-to-all connections between PFC and feature-selective neurons are not feasible. Moreover, how can we then explain studies that *do* demonstrate decoding of WM from prefrontal areas? In reality, we envisage that each conjunctive neuron is likely to receive inputs from only a subset of feature neurons. In order for conjunctive neurons to bind features into objects, these inputs must at least include multiple feature dimensions *and* multiple features in each dimension. The model is therefore potentially compatible with the presence of mixed selectivity (Rigotti et al., 2013), which would provide a background of weak input selectivity based on the presence or absence of connections, upon which rapid plasticity is superimposed. This means that the variable selectivity predicted by the model (Fig. 5) would not be as strong in real neurons, and indeed single-unit studies do suggest considerable stability in monkey prefrontal cortex, at least when averaged over many trials (Freedman et al., 2001; Rainer et al., 1998). We note that stable mixed selectivity, even without plasticity, could in some situations produce binding and capacity limits (Matthey et al., 2015). However without additional mechanisms, it would presumably not account for attentional shifts, activity-silent storage, or apparent control over posterior cortical areas, and moreover it complicates many accounts of how other brain areas would ‘read-out’ WM contents.

Further, there may also be significant topography in conjunctive cells connectivity, which we have neglected. For example, different regions of prefrontal cortex may be specialized for remembering different kinds of information (Romanski, 2004). This may have two desirable consequences. First, aspects of the attended object – especially information that is highly topographical in posterior areas, such as stimulus category and spatial location – would be consistently decodable from prefrontal cortex (Lee and Baker, 2016) but will be modulated by relevance (Kornblith and Tsao, 2017). Second, conjunctive neurons in different prefrontal subregions may connect preferentially to visual, motor or auditory cortex, which could account for the separability of visuospatial and phonological WM and also their overlap (Morey et al., 2011). One concern with freely-conjunctive neurons is that, in order to allow truly arbitrary information or instructions to be stored, they would need an implausibly large array of hard-wired inputs. These concerns might be allayed by including topography, e.g. intermediate layers of flexible but more domain-specific conjunctive neurons.

Some studies show that more than one item can be prioritized and recalled better. Does this indicate that attention can highlight more than one item at a time (Cowan, 2011)? Some recent findings suggest there may be two or more “foci of attention” (Christophel et al., 2018; Sutterer et al., 2018). Our model may still be compatible with some of these findings, as it predicts graded benefits for more than one item. Recently-focused items have facilitated synaptic weights, even though only one item is technically held in an active state. An alternative strategy might be to directly permit multiple foci of attention within this model. To do this, conjunctive neurons could be partially segregated, which in theory could generate more than one focus of attention (although this would require reduction of inhibition between features, and would significantly disrupt encoding).

We treated “features” as just simple perceptual attributes, but we believe that our class of feature-selective neurons could include any aspect of the world that is encoded in a stable way, including those aspects that incorporate long-term knowledge, such as object identity, category, or even linguistic information such as word meanings. These attributes are likely to be encoded stably in posterior cortical areas, in contrast to the temporary combinations of information represented in an ephemeral way – e.g. for online manipulation – as typified by our conjunctive neurons. The current simulations used only a single, rapid learning rate, but it remains to be studied how this could be reconciled with longer-term learning.

Biological sensory neurons encode perceptual features on a continuous domain, with overlapping neural selectivities; however the present model used only a few discrete features. It would therefore be important to confirm that our model could also be extended to continuous feature domains, to predict the range of proximity phenomena accounted for by other models (Oberauer and Lin, 2017). Moreover, unlike our model, visual representations in posterior cortex are arranged spatially, such that space is a fundamental component of all other feature representations. In its present form, our model does not account for the unique role of space in visual WM (Pertzov and Husain, 2014; Wang et al., 2016), but we argue that its simpler form better explains how a single architecture could hold generic, content-general information in WM.

### 3.4 Internal control over attentional shifts

We have assumed that attentional shifts are externally cued. Endogenous shifts of attention are not modelled. One way of implementing internally-generated attentional modulation would be to de-stabilize the persistent activity by adding delayed suppression, or refractoriness, to the competitive conjunctive neurons. The result would be that, after an object is attended, its activity is extinguished after a delay, leading to a transient and unstable focus of attention. Akin to some models of visual attention guidance (Itti and Koch, 2001), attention may then be successively re-deployed towards weaker-represented features in WM. This would be needed to account for three key phenomena: (a) rehearsal, in which attention moves sequentially between items during a memory delay, (b) the ability to free-recall WM items in order, and (c) to permit serial encoding of a simultaneously-presented memory array. Our model currently relies on each object to be presented or attended sequentially, like the temporal context model (Howard and Kahana, 2002).

Although WM maintenance commonly engages PFC, evidence from neuropsychology and functional imaging suggests PFC’s role includes cognitive control, WM manipulation, and response selection, rather than simply WM storage (Bechara et al., 1998; D’Esposito and Postle, 1999; Rowe et al., 2000; Thompson-Schill et al., 2002), and it remains to be tested whether the conjunctive neurons we propose can perform such functions. For example, we cannot account for the ability to “gate out” distractors, and prevent them from being encoded in WM. How could *irrelevant* distractors be ignored, while still allowing relevant inputs to capture attention? To achieve this, conjunctive units would themselves need to be under higher-level control. The current model, with only one layer of conjunction units, does not explain higher order control of attention, since sufficiently-strong bottom-up stimuli that match a conjunction will always tend to re-activate that conjunction and thus capture the focus attention. The conjunction and feature neurons together simply act as a “matched filter”, amplifying patterns that have recently been active (Chrysikou et al., 2014; Hayden and Gallant, 2013). The model also cannot yet perform *n*-back tasks, where a decision must be made regarding items presented earlier in a sequence. After an item is presented, attention seems to shift back to previously-presented items (Greene et al., 2015). Perhaps gating vs granting access to working memory by preventing this might be controlled by interactions between prefrontal cortex and the basal ganglia (Badre, 2012; Chatham et al., 2014).

In some studies, activity-silent representations have been associated with so-called non-conscious WM. In this phenomenon, the identity of a subliminal masked stimulus can be guessed above chance despite reports that no stimulus was seen, and despite an intervening distractor (Soto et al., 2011). This non-conscious storage has been accounted for by storage in synaptic weights (Trübutschek et al., 2017), in line with non-conscious episodic memory (Chong et al., 2014). This contrasts with our model, however, in which information in synapses can be fully reactivated and brought back into the focus of attention, and would thus presumably be reportable.

### 3.5 Location of conjunctive neurons and their plasticity

Conjunctive-coding neurons might not be confined to prefrontal cortex. Other regions that play a role in working memory, such as the hippocampus, basal ganglia or thalamus, might also contain freely conjunctive neurons. Moreover, there may be a continuum or overlap of mechanisms subserving working memory and episodic memory (Fiebig and Lansner, 2014). Quite unlike the long-lasting episodic encoding proposed in the hippocampus, however, the volatile synaptic weights we propose would produce strong but evanescent trial-by-trial selectivity changes (Fig. 5). A more intriguing possibility is that both freely-conjunctive and stable-feature neurons are actually present in the *same* brain regions, with a spectrum between highly-plastic and stably-coding neurons.

The Hebbian rule we use could share mechanisms with long-term potentiation (LTP). Stimuli too weak to elicit LTP can still elicit synapse-specific potentiation that decays over minutes, sometimes termed “short-term potentiation” or “relatively short-lasting LTP” (Malenka and Nicoll, 1993; Frey and Morris, 1998). This potentiation differs from post-tetanic potentiation and short-term facilitation (Jin et al., 2011) in that it includes a postsynaptic component (Huang et al., 1992). This kind of rapid-onset, postsynaptic-dependent plasticity is sufficient for our WM model to operate (Fig. S11), irrespective of whether it decays over minutes or not. Fewer studies have quantified postsynaptic potentiation in PFC, but those that have show similar effects: a single 500 ms train produces 130% facilitation lasting 20 min, which is NMDA dependent and modulated by dopamine (Huang et al., 2004).

In summary, a single architecture captures both persistent activity attractors and silent synaptic memory. We introduce a new scheme of transient flexible neuronal coding, that can support many empirical phenomena (Tables S1/2) including the “focus of attention”, and generates numerous testable neural predictions.

## Footnotes

Appendix A Supplementary material related to this article can be found, in the online version, at doi:https://doi.org/10.1016/j.neubiorev.2019.03.017.
