# Supplementary material for: Neural mechanisms of attending to items in working memory
Source: Neurosci Biobehav Rev. 2019 Jun;101:1–12. doi: 10.1016/j.neubiorev.2019.03.017 (PMC6525322; doi:10.1016/j.neubiorev.2019.03.017)
Supplement: Supplementary file 1 [file mmc1.pdf]

Neural mechanisms of attending to items in working memory

Supplementary Materials:

Methods

- Implementation of stimuli and simulations
- Simulations 1 to 24

    - Simulation 1: Set size reduces accuracy
    - Simulation 2: Serial position shows primacy and recency effects
    - Simulation 3: Encoding duration
    - Simulation 4: Memory delay results in increasing interference over time
    - Simulation 5: Transposition errors
    - Simulation 6: Incidental retrocue benefit
    - Simulation 7: Reaction time (RT)
    - Simulation 8: The probe itself can interfere with recall
    - Simulation 9: Weak TMS pulse reactivates delay period decoding
    - Simulation 10: Strong TMS pulse disrupts focus of attention
    - Simulation 11: Delay-period decoding of focused item
    - Simulation 12: Previous trial repetition benefit
    - Simulation 13: Pattern similarity after intervening conjunctions
    - Simulation 14: Implementing simple task rules
    - Simulation 15: Removing the third (task-irrelevant) feature dimension
    - Simulation 16: Exploring the parameter space
    - Simulation 17: Non-Hebbian (presynaptic) plasticity
    - Simulation 18: Plasticity in feature-to-conjunction synapses only
    - Simulation 19: Feature-to-feature plasticity without conjunctive neurons
    - Simulation 20: Synaptic decay time must be at least minutes
    - Simulation 21: Inverted neural representation of unattended items
    - Simulation 22: Increasing the number of conjunctive neurons
    - Simulation 23: Repeated features on the irrelevant dimension
    - Simulation 24: Retrieval of whole objects vs. individual features
- Minimal algorithm

Figures and Tables

- Figures S1-S17
  - **Figure S1:** Reaction times (Simulation 7)
  - **Figure S2: Novel prediction** – Probe interference (Simulation 8)
  - **Figure S3**: Errors are more likely to be sequentially neighbouring items (Simulation 5)
  - **Figure S4**: Decoding from feature units during the delay period (Simulation 11)
  - **Figure S5**: **Novel prediction**: Trial-to-trial conjunctions effect (Simulation 12)
  - **Figure S6**: Acting on multiple task rules (Simulation 14)
  - **Figure S7: Novel prediction:** Benefit with an extra feature dimension (Simulation 15)
  - **Figure S8**: Influence of model parameters upon central behavioral effects (Simulation 16)
  - **Figure S9:** Basic results for 'high-accuracy' parameter regime (used for simulations 3 to 5)
  - **Figure S10**: Failure of simplified versions of the model (Simulations 17, 18, 19)
  - **Figure S11**: Allowing decay of synaptic weights (Simulation 20)
  - **Figure S12**: Cross-decoding reveals inverse coding of unattended items (Simulation 21)
  - **Figure S13**: Decay effect during the delay period split by serial position
  - **Figure S14**: Increasing the number of conjunctive units to 8 (Simulation 22)
  - **Figure S15**: Estimated precision follows power-law with set size (Simulation 1)
  - **Figure S16: Novel Prediction**: Effects of repetition of irrelevant features across items (Simulation 23)
  - **Figure S17: Novel Prediction**: Correlated and uncorrelated recall of two features of the same objects (Simulation 24)
- **Table S1** Empirical findings explained by the model
- **Table S2**: Psychological concepts corresponding to the model
- Movie S1 legend

Supplementary References

Methods

The present model considers a minimal arrangement for three feature dimensions, each with four possible feature values, allowing 12 features to be encoded. Each feature unit receives input when a particular feature is present in the stimulus. Four conjunctive units are fully connected reciprocally to the 12 feature units. These connections are all excitatory, and initialized to be random. Four conjunctive neurons is the minimum possible number that can bind information from four objects. The fully-connected network therefore required 48 weights to conjunctions from features ($\mathbf{W}^{cf}$) and 48 to features from conjunctions ($\mathbf{W}^{fc}$). The activity of all units $\mathbf{f}$ and $\mathbf{c}$ were initialized to zero at the start of simulation, and weights $\mathbf{W}^{fc}$ and $\mathbf{W}^{cf}$ were randomly assigned from a uniform distribution over the interval [0, 1]. In its simplest form, the activity update equation was:

$$\begin{bmatrix}\mathbf{c}\\ \mathbf{f}\end{bmatrix} \leftarrow \mathrm{sigmoid}\left(\begin{bmatrix}\mathbf{W}_{cc} & \mathbf{W}_{cf}\\ \mathbf{W}_{fc} & \mathbf{W}_{ff}\end{bmatrix}\begin{bmatrix}\mathbf{c}\\ \mathbf{f}\end{bmatrix} + \begin{bmatrix}\mathbf{0}\\ \mathbf{input}\end{bmatrix}\right)$$

The conjunctive-feature synapses $\mathbf{W}^{cf}$ and $\mathbf{W}^{fc}$ were updated by a Hebbian covariance rule, whereas the fixed inter-conjunction ($\mathbf{W}^{cc}$) and inter-feature ($\mathbf{W}^{ff}$) synapses each comprised two components:

1) blanket lateral inhibition between conjunction neurons or between features within the same dimension, to implement competition, and
2) self-excitation, so that firing does not stop suddenly when external input is removed, but rather decays exponentially with time.

So the update equations can be written out more fully as:

neuron activation:

$$\mathbf{c} \leftarrow \sigma(\beta + (\alpha_1 \mathbf{1} \quad\quad + \alpha_2 \mathbf{I})(\mathbf{c} - \beta) + \alpha_3 \mathbf{W}^{cf}(\mathbf{f} - \beta) + \varepsilon \cdot \mathcal{N})$$
$$\mathbf{f} \leftarrow \sigma(\beta + (\alpha_4 \mathbf{W}^{ff} + \alpha_4 \mathbf{I})(\mathbf{f} - \beta) + \alpha_6 \mathbf{W}^{fc}(\mathbf{c} - \beta) + \mathbf{i})$$

synaptic updates

$$\Delta = (\mathbf{c} - \beta) \cdot (\mathbf{f}^T - \beta)$$
$$\mathbf{W}^{cf} \leftarrow \sigma(\mathbf{W}^{cf} + \gamma_1 \Delta)$$
$$\mathbf{W}^{fc} \leftarrow \sigma(\mathbf{W}^{fc} + \gamma_2 \Delta^T)$$

constants:

$$\alpha_i = \{-0.28, 1.03, 0.05, \ -0.28, 0.75, 0.05\}$$
$$\beta = 0.175$$
$$\gamma = \{0.02, 0.02\}$$
$$\varepsilon = 0.005$$
$$\mathbf{W}^{ff} = \begin{cases} 1, & \text{if } i, j \text{ are in the same feature dimension} \\ 0, & \text{otherwise} \end{cases}$$
$$\sigma(x) = \begin{cases} < 0 : & 0 \\ 0 \leqslant x \leqslant 1 : & x \\ > 1 : & 1 \end{cases}$$
$$NC = 4$$
$$NF = 12$$

In these equations, **c** and **f** are the activities of conjunctive neurons and feature neurons, and **i** is the external input. The six synaptic free parameters α were:

α_1 , α_4 : mutual lateral inhibition between neurons

α_2 , α_5 : self-excitation or temporal decay, and

α_3 , α_6 : synaptic gain for the conjunction-to-feature and feature-to-conjunction synapses,

for the conjunctive and feature neurons respectively. β is the baseline neuron activity, γ_i are the learning rates, and ε is the amount of noise added to the conjunctive units. The function σ constrains values lie between 0 and 1 and, for simplicity, was chosen as: $\sigma(y)=\min(1, \max(0, y))$. The identity matrix **I** produces self-excitatory synapses, and a matrix of ones (**1**) produces lateral inhibition. In simulations, the 12 features were arranged into 3 dimensions (color, orientation and location), so that the feature-to-feature inhibition ($\mathbf{W}^{ff}$) was arranged in 3 blocks of 4 units. N indicates a gaussian white noise vector with s.d.=1. We constrained learning rates to be identical in both directions ($\gamma_1=\gamma_2$), and noise was in fact not required in order to obtain the typical patterns of errors reported here ($\varepsilon=0$), giving effectively 8 free parameters.

Note that the plasticity rule we employ in the model has no explicit temporal decay; because capacity limits are generated through interference, we only require that the plasticity lasts longer than the memory delay. A minimal algorithm to reproduce the main result is provided at the end of this section.

Implementation of stimuli and simulations

Model equations were simulated in MATLAB (code available at [http://www.smanohar.com/wp/wm/download.html]). A web-based interactive simulation can be accessed at http://www.smanohar.com/wp/wm. Each run simulated 200 trials. To test different hypotheses, the simulation setup was varied from a canonical setup. The canonical simulation resembled common working memory experiments (Bays and Husain, 2008). A series of objects was presented, followed by a memory probe that triggers recall. Each object excited one feature in each dimension (colour, orientation and location). The features of the objects presented on each trial were random, with the constraint that no two objects within one trial shared any

features. Each trial started with a foreperiod for equilibration lasting 200 time steps, with the features inhibited (input **i** = –1). Then during the encoding epoch, each memory item was presented by activating the corresponding feature units, by maximally activating the features present in the object (i = +1) and inactivating the absent features (i = –1). Stimuli were presented for 120 time steps, followed by an inter-stimulus interval of 50 time steps with no input (**i**=0). After the last item was presented, the memory delay period of 240 time steps followed, with no input, mimicking a retention interval. Then at the probe epoch, the feature acting as the retrieval cue was activated (i=+1), and the other features were inhibited (i = –1). The probe lasted 120 time steps. Finally, the response interval constituted 220 time steps with no input. This permitted re-activation of the features to be recalled. The feature that reached the highest level of activity during this period was selected as the response. In the case of an exact tie-breaker the decision was randomized. An example of this sequence of events shown in **Video S1**.

By setting feature values to -1 during the foreperiod, this extinguishes the focus of attention of the previous trial, corresponding to the idea that participants might be thinking about something different between trials. Since we had no other feature neurons to represent non-task information, we wanted to manually inactivate the feature units corresponding to the current trial, consistent with attention shifting away from the previous response. If the inhibition were not sustained through the foreperiod, one of the conjunctive neurons would become active again due to the excitatory-inhibitory balance, to re-activate one of the items from the previous trial. The effect of the inter-trial inhibition is two-fold: first it weakens the synaptic weights present from the previous trial, and second, the first item of the new trial does not need to de-activate a previously

active item in the focus of attention. This is the source of the primacy effect, and removing the inhibition abolishes the primacy effect.

Parameter selection was performed initially by trial and error to achieve the hypothesized dynamics. First, the decay, inhibition and input weightings α_i for the conjunctive and feature neurons were adjusted to create a sustained activity plateau and to ensure that conjunctive neurons competed in a winner-takes-all manner. Then the learning rates γ_i were adjusted to allow sufficiently rapid weight changes such that over 50 time steps, a trace remained that represented which combination of feature units had been active. The baseline was approximately 20% maximal (i.e. $\beta = 0.2$) to permit deactivation of neurons, so that conjunction units that lose the competition will ‘unlearn’ their associations (Stanton and Sejnowski, 1989). A small amount of noise was added to ensure conjunction units were never identical, to facilitate symmetry-breaking.

Certain effects were more or less sensitive to changes in model parameters. Overall accuracy could be made to range from consistent 100% to consistent 0%, depending on choices of α_i, β, γ_i and ε. We aimed for 70% overall accuracy, allowing a wide dynamic range of performance to examine the predicted effects, so this was the regime in which the main simulations were run.

Simulation 1: Set size reduces accuracy

Simulation parameters were as above, with $\alpha_i = \{ -0.28, 1.03, 0.05, \ -0.28, 0.75, 0.05 \}$; $\beta=0.175$, $\gamma = \{0.02, 0.02\}$; $\varepsilon = 0.005$. Timings were: foreperiod=200 steps, presentation durations of stimuli at encoding = 120 steps, inter-stimulus = 50 steps, retention delay=240 steps,

probe=120 steps, recall=240 steps. Trials proceeded as above, with four items presented sequentially. One, two, three and four items were presented in different trials, and each serial position was probed. For multi-item sequences, each item in the sequence was probed equally often. This gave ten (1+2+3+4) trial types, and 200 trials of each type were simulated. The average accuracy over all serial positions was calculated for each set size (**Fig.2B**). Error bars are the standard error of the accuracy when trials were broken into subsets of 20 trials each. Simulations were performed both using interleaved trials, and also with one condition per block, and comparable results were obtained with both methods.

Capacity limits were naturally limited to four in this simulation because only four conjunctive neurons were present. However the capacity limit does not *directly* relate to the number of conjunctive neurons, but rather, to the *proportion* of conjunctive neurons that will become simultaneously active during winner-takes-all competition (¼ in this case). This is in turn determined by the level of inhibition. A simulation using 8 conjunctive neurons also reproduced the set-size effects, with inhibition tuned to allow two neurons to be active at once.

Note that absolute modelled accuracy was lower than in the empirical data, because participants made same/different judgements (chance=50%) whereas our simulations recalled actual features (chance=25%). To compare our results with the power law observed for precision (Bays & Husain, 2008), a surrogate for precision was estimated, because our simplified network imposes no metric structure over features. This was calculated by assuming nontarget responses were evenly distributed over a circular domain, and taking the circular standard deviation (**Fig.S15**). The more errors, the higher the s.d., and the lower the precision. A power law curve was fitted to

the precision as a function of the set size $P = N^{-k}$, where P is precision, N is the set size ,and k the fitted exponent. This yielded $k = 0.76$, comparable to the empirical value $k = 0.74 \pm .06$.

Simulation 2: Serial position shows primacy and recency effects

The run was identical to simulation 1 above. Trials were grouped according to the serial position of the probed item, and by set size (**Fig.2D**). Accuracy was calculated as per simulation 1. Note that in all simulations, feature unit activity was held at zero at the start of each trial (**i** = –1) to simulate a foreperiod or inter-trial interval. This permitted the first item of the sequence to be encoded faster. We then explored different parameter sets, varying α, β, γ and ε. The recency effect was robust across a wide range of parameters, and was often strong enough to push performance to 100% for the last item. The presence of a primacy effect was more strongly dependent on the specific presentation timings and on the choice of α_i . This matches empirical studies of visual WM, which do not always find primacy effects.

Simulation 3: Encoding duration

The time that each item was presented for was varied. This ranged from 10 to 120 time steps (in increments of 20 ms), with all items being presented for the same duration. The inter-object duration was fixed at 50 ms as in previous simulations. The average accuracy was collapsed across all serial positions for each set size, and plotted as a function of encoding duration (**Fig.2F**). The simulation demonstrates an interaction between set size and encoding time, such that when more items are stored, the asymptotic information about each object is lower, and the rate of encoding is proportionately slower. In order to avoid floor effects for this simulation, where accuracy in the 4-item condition could approach chance very quickly as the encoding

duration decreases, we increased the 'stickiness' of conjunction units in the model, by reducing the decay factor α_1 and uptitrating their input gain from each other (α_2) and from the feature units (α_3). We therefore set

$$\alpha = \{ -0.5, 1, 0.08, \quad -0.28, 0.7, 0.05\} \quad \text{and} \quad \beta = 0.2$$

for this simulation, and the consequence was that the overall accuracy of the model increased from 75% to around 90% while preserving the set size and serial position effects. This 'higher performance' regime was used for simulations 3, 4 and 5. Other than improved overall accuracy, these parameters produced qualitatively similar effects to the primary simulations (**Fig.S9**).

Simulation 4: Memory delay results in increasing interference over time

We varied the number of time steps after the final item was presented, until the onset of the probe. The delay varied between 200 and 1800 steps. For each duration, simulations of 200 trials were run for 1, 2, 3 and 4 items, with each possible position being probed (i.e. total 2,000 trials per duration). In order to avoid floor effects, where accuracy in the 4-item condition could approach chance as the delay increases, we used the same regime as Simulation 3. Data is plotted as a function of the delay (**Fig 2H**). The simulation demonstrates an interaction between set size and time, such that when more items are stored, the decay is faster.

Simulation 5: Transposition errors

Here we studied the tendency to incorrectly report features from items temporally adjacent to the probed item. This arises because occasionally, the same conjunctive unit is activated for two consecutive objects, when the second object fails to sufficiently drive a different conjunction unit. In this case, features of two consecutive objects will be confused.

Data were taken from the standard 4-item condition where four items were presented and one is probed, using parameters of Simulation 3. For this simulation, trials were grouped according to serial position of the probed item. Four responses were possible on each trial, and for each trial, we take the serial position at which the reported feature *actually* appeared on that trial (**Fig.S3B**). This figure shows a histogram of the model's responses. Over 2000 trials, the probability of making each of the four responses was calculated. Each line shows the probability of reporting the orientation of the four items presented in the sequence (x-axis), when a particular serial position was probed (each as a different line). We show logarithms of the mean error rate as in (Farrell and Lewandowsky, 2004), and added a small offset of 10^{-3} since some runs had zero errors. Error bars are standard error of the logarithm of mean error rate. The four possible responses are aligned such that the correct response appears at position zero, at the center of the graph (i.e. the *probed* item's orientation). Sometimes the model erroneously reports an item previous to the one probed ($x<0$) or an item later than the item probed ($x>0$). The mean of the four probe conditions is shown in red.

Simulation 6: Incidental retrocue benefit

In the empirical study, the primary task involved recalling the orientation of the item with a given color, and the secondary task ("incidental cueing") required participants to report the location of the item with a given color, which could be congruent or incongruent to the ultimately-probed item. We simulated the empirical task (**Fig.3D**) by presenting two items, as per the 2-item condition in simulation 3. After the items were presented, a 120 time-steps retention period followed, then the probe feature (incidental cue) for one of the two items was activated for 40 time steps ("IC" in **Fig. 3E**). This was achieved by activating the cued colour feature ($i = +1$) and inactivating the other three colour features ($i = -1$). After a further 120 time

steps corresponding to recall of the third dimension for this item, a further memory delay of 120 time-steps was included. The total delay was therefore 280 timesteps. Then the final memory probe was activated, and recall of the second dimension was measured as previously. This final probe could either be the same item (congruent), or the other item (incongruent), as the one cued for the first response. 200 trials were simulated for each condition. Accuracy was plotted for the valid and invalid incidental cue conditions (**Fig. 3F**).

Simulation 7: Reaction time (RT)

RT was calculated by finding the time at which the winning feature reached its maximal value in the period after the probe. The time to reach 98% of maximum was used, rather than using an absolute threshold, because the final stable value of an activated feature differed when different model parameters were used. Using an arbitrary fixed threshold or the rate of rise yielded qualitatively similar but less consistent RT effects. To examine basic set size and serial position effects, the same trials were used as in simulation 1. Mean RT with standard error is shown (**Fig.S1A**), in comparison to data (McElree and Dosher, 1989) (**Fig.S1B**). RT was generally inversely related to accuracy.

Simulation 8: The probe itself can interfere with recall

Many working memory tasks have asked participants to adjust features of the probe to match the remembered features (Zhang and Luck, 2008). In these experiments, the probe contains a feature that is irrelevant to recalling the item. For example, if participants must report the orientation of a bar with a given color, then using a colored bar as a probe introduces an orientation feature that conflicts with the remembered item. The model predicts this will interfere with re-focusing the item (**Fig.S2**). To simulate this, sequences of 1 to 3 items were presented to the model, using an

identical setup to simulation 1. However at the time of probe, two features were activated; one in the probe dimension, and one in the recall dimension. As previously, the probe feature input was +1, and other features on that dimension were -1. However an additional input +1 was added for one orientation feature. The additional feature, in the recall dimension, was one that had *not* been presented on that trial. Since only 4 features were present in this model, this constraint meant that we could only test set sizes of one to three items.

Performance was worse when the probe contained an interfering item. There is some empirical support that this might indeed be the case (Souza et al., 2016). The simulation predicts interference across all set sizes. Conversely, we can also predict an improvement in performance for probes containing an additional *helpful* feature, for example if participants must report an object's orientation given both its color *and* its location.

Simulation 9: Weak TMS pulse reactivates delay period decoding

Here we enquired whether the model could reproduce the phenomena where unattended items, which are not normally decodable from brain activity, could be brought back into a temporarily decodable state by applying a pulse of activation. Empirically this has been demonstrated using a nonspecific high-energy visual stimulus pattern (Wolff et al., 2017), or by applying a TMS pulse to sensory cortex (Rose et al., 2016). In the simulation, two items were presented sequentially to the model, exactly as in simulation 1. In the middle of the delay period, the feature neurons received a flat high-valued input (**i**=+1) for 10 time steps. This was compared to an identical condition with no stimulus. The delay period therefore comprised either 120 timesteps + 10 timestep pulse + 120 time steps, or in the no-stimulus condition, 250 timesteps (**Fig 4A**).

2000 trials were simulated, and half the trials were used to construct a linear classifier that predicts the identity of each of the two items presented on that trial. The two classifiers were

tested on the remaining trials, to give the decoding accuracy, using lclassify function in Matlab. Decoding was performed across trials at each time point independently, indicating the degree to which neural activity in the feature units predicted the identity of each item. The first object presented was termed the 'unattended' object, since during the delay, attention was focused on the second (final) object. Decodability of the unattended item was transiently restored after the pulse (**Fig.4B** dark blue trace), reproducing the phenomenon in the TMS study (**Fig.4C**) and using a high-energy neutral visual stimulus.

Two further predictions are that 1) stimulation of prefrontal regions should have similar effects, and 2) selectively stimulating specific feature neurons e.g. in sensory cortex will have stronger effects when those features are part of an item currently in memory – i.e. when the synapses from that feature neuron to the conjunctive neurons are already strong.

Simulation 10: Strong TMS pulse disrupts focus of attention

A strong TMS pulse to sensory cortex (MT+) has been shown to disrupt the focus of attention. To reproduce this, we used precisely the same simulation as in 9, but increased the pulse duration to 20 timesteps duration. We compared accuracy for probing the first item, vs the second item that was presented, with and without pulse. The presence of the pulse reduced accuracy for the second item, but paradoxically improved memory for the first item (**Fig.4D**). This is because the pulse indiscriminately re-activated the feature and conjunction neurons, disrupting the focus of attention but not the underlying memory traces. Indeed after the pulse, the attractor state sometimes shifted back to the first item. Precisely this phenomenon was observed in a TMS study (Zokaei et al., 2014a) (**Fig.4E**). The model predicts the same effects if prefrontal neurons are stimulated.

Simulation 11: Delay-period decoding of focused item

Three items were presented in sequence to the model. This was identical to simulation 1 except that the delay period duration between items was increased to 100 time steps, to test decoding during the stable attractor state. Each serial position was probed on 2000 trials. As in simulations 9 and 10 above, half the trials were used to construct linear classifiers that predict the identity of the item that was shown at each given serial position on a trial. The three classifiers were then tested on the remaining trials, to give the decoding accuracy. Decoding at each moment in a trial indicates the degree to which neural activity in the feature units predicted the identity of the item presented at each serial position (**Fig.S4A**). We then asked 6 questions, as in (Konecky et al., 2017): can the first item be decoded in the first delay, in the second delay, or in the third delay; can the second item be decoded in the second or third delay; can the last item be decoded in the third delay (**Fig.S4B**)? The feature neurons strongly encoded the most-recently-presented item. Using the activity of conjunction units, however, nothing could be decoded above chance using a linear classifier.

Simulation 12: Previous trial repetition benefit

This effect was examined by using all trials taken from simulation 1. We compared trials in which the probed-item's features on the probe dimension and the recall dimension were the same or different to the probed item of the previous trial. Trials were grouped according to whether the previous trial's probe color was the same as the current trial's probe color, and also whether the probed-item's orientation was the same or different to the previous trial's probed-item orientation (**Fig.S4**). When the identical item was probed, recall was more accurate.

Simulation 13: Pattern similarity after intervening conjunctions

We ran 2000 trials of the 1-item memory condition, and examined delay-period activity in conjunction units. If neurons have classical receptive fields, then when the same stimulus is presented as on a previous trial, the activity pattern will be similar, whereas if the stimulus is different, the patterns will be dissimilar. Two major predictions of flexibly-conjunctive neurons is that the pattern similarity will decrease both with the number of intervening trials (**Fig.5A**), and also when intervening stimuli form different conjunctions with the same features (**Fig.5B-D**).

The similarity between representations on trial *n* and trials *n*-2, *n*-3 up to *n*-10 were examined, in the middle 100 timesteps of the delay period. For each inter-trial distance, trials were divided according to whether the same or different stimulus was shown on those two trials (**Fig.5A**). We considered only 2 feature dimensions in this analysis, so that each pair of trials had the same stimulus 25% of the time. For *n*-1 to *n*-4, it was possible to further subdivide trials according to the stimuli presented on intervening trials. A "violation" of the conjunction was defined as an intervening stimulus which is similar on one feature dimension but dissimilar on the other feature dimension, to trial *n*. No violation occurs if the intervening stimulus is the same as on trial *n*, or if it contains no features in common with trial *n*. Trials were split according to the number of these intervening violations.

Simulation 14: Implementing simple task rules

To implement execution of actions, we re-labelled the 'orientation' dimension as 'action', so that there were 4 possible motor actions, which could be coupled to the 4 possible colors (**Fig.S6**). To provide the model with instructions for the task rules, each stimulus-response (S-R) mapping was activated, one at a time. For example, to provide the instruction "red means press button 1", we activated the "red" colour unit and the "button 1" action unit simultaneously. Analogous to

working memory encoding, a conjunction unit became associated with each pairing. In each block, we presented either 1, 2, 3 or 4 rules. After presenting the rules, 12 color cues drawn equiprobably from that set of rules were shown sequentially in a randomized order, analogous to memory probes. After each cue, activation of the motor units was measured. We tested the responses to one, two, three and four simultaneous S-R mappings.

The sequence of events was thus very similar to the working memory task, and reaction times and accuracy were calculated in the same way as before (**Fig.S6A**). Two differences in implementation were needed to permit appropriate action selection over many trials. First, we lengthened encoding (240 steps) and shortened the probe duration (80 steps). This increased the stability of the mappings. Second, rather than inhibiting the features during the inter-trial interval, no input was provided (**i** = **0** rather than **-1**), which allowed the units to maintain their ongoing activity. The attentional focus thus remained active throughout the experiment. Without these two changes, interference led to forgetting of the task rules over the first 5 to 10 trials.

Two important empirically observed effects in choice reaction time experiments are Hick's law – the increase in RT with log(number of options) – and the repetition effect. To examine Hick's law, we calculated the mean RT on correct trials, in blocks where there were 1 to 4 rules presented (**Fig.S6B**). To study the effect of stimulus-response repetition on consecutive trials, trials in the 2-rule blocks were categorized according to whether the same stimulus was present on the previous 0, 1 or 2 trials (**Fig.S6C**), and the mean RT was calculated separately for correct and incorrect response trials. Data for correct trials from (Schvaneveldt and Chase, 1969) were re-plotted next to simulations. Decoding from conjunctive units was performed as previously

done for feature units, as a function of time. For each trial the classifier was trained on other trials in the same block (**Fig.S6D**). For comparison, accuracy when the classifier was trained on the previous block was also measured. During WM tasks no decoding was possible from conjunctive units. But in this simple stimulus-response task, decoding was possible across a block of trials which shared the same rule. The presentation of new task rules at the start of a block effectively overwrites some of the conjunctive neurons' weights, attenuating the ability to decode the stimuli across rule changes. Note that there is still residual decoding from the previous block, because for several rule changes there are shared stimulus-response pairings. Indeed there is a 1 in 4 chance that any stimulus can re-use the same conjunctive neuron as on the previous rule, without overwriting its synaptic weights. Note one idiosyncrasy of the leave-one-out method on short shuffled blocks of trials is that we obtain below-chance prediction of the upcoming stimulus before presentation. The shuffling means that on average, in 6 out of 11 of the other trials in the block, residual activity from the previous trial predicts the wrong upcoming stimulus.

Simulation 15: Removing the third (task-irrelevant) feature dimension

In previous simulations, each object consisted of three features, but one of the features is task-irrelevant. Simulation 3 was run with standard conditions and timings but on half of trials, the third dimension features were treated as absent (**i**=-1) for every object. We contrasted recall accuracy for conditions where objects consisted of all 3 features vs. only 2 features (**Fig.S7**). The primacy and recency effects were observed to be smaller.

Simulation 16: Exploring the parameter space

We examined 9 free parameters in the model: the baseline activity, lateral inhibition x 2 (for conjunction and feature units), self-excitation x 2, reciprocal excitation x 2, and learning rates x 2 (**Fig.S8**). For each combination of parameters, 50 trials per condition were simulated for set-sizes 1 to 4, for all serial positions (500 trials per parameter set). The canonical model was perturbed along two parameters at a time. For each pair of parameters, a range of values above and below the canonical parameter values were tested, with 10 linearly-spaced levels of each parameter, to give a 10x10 grid. The parameter values used in Simulation 1 thus lie at the center of each grid, and the figure represents all possible cardinal planes through that point in a 9-dimensional hyperspace.

Each simulation resulted in a serial position curve like Fig.2D, from which we could quantify the set size effect (linear slope of accuracy as set size varies, collapsed across serial position), recency effect (difference in accuracy between final and penultimate items in sequence, averaged across set sizes 2-4) and primacy effect (difference in accuracy between first and second items in sequence, averaged across set sizes 2-4). The size of each effect was smoothed using a 3x3 boxcar, and is portrayed by pixel color in the 10x10 grids.

Simulation 17: Non-Hebbian (presynaptic) plasticity

For these simulations, the Hebbian synaptic rule was replaced by a simple rule that dependent solely on the presynaptic neuron activity. The model was run both with long-term non-decaying weights, and weights that decayed with a half-time of 20 steps. The update rules were

$$\Delta\mathbf{W}^{cf} \leftarrow \gamma\mathbf{f}\cdot\mathbf{1}^{T} - \kappa\mathbf{W}^{cf}$$
$$\Delta\mathbf{W}^{fc} \leftarrow \gamma\mathbf{c}\cdot\mathbf{1}^{T} - \kappa\mathbf{W}^{fc}$$

Where γ is the learning rate, $\boldsymbol{\kappa}$ is a decay constant for the synaptic weights, and the $\mathbf{1}^T$ indicates that all the synapses of the presynaptic neuron are modified together. We used learning rate γ =0.02, and plasticity decay κ ranging from 0.02 (20 timesteps) to 0.0001 (5000 timesteps). In our small all-to-all network, it was not possible to obtain stable persistent activity with this regime of plasticity (**Fig.S10A**). This was because the facilitation was not synapse-specific, resulting in just a generalized increase in activity of the target neurons. Thus no parameter combination would permit feature binding, with this architecture.

If a large number of conjunctive neurons with pre-wired connectivity were employed, such that each conjunction neuron has selective synapses both to and from the same subset of feature neurons, then there exist regimes that can support persistent activity, and the network operates more or less like previously described STP-based WM (Mongillo et al., 2008).

Simulation 18: Plasticity in feature-to-conjunction synapses only

In this simulation we abolished plasticity from the conjunction neurons to feature neurons. Thus plasticity was limited to just the feature-to-conjunction direction. $\mathbf{W}^{cf}$ was randomly initialised as before but thereafter fixed.

In this network, sustained activity was in fact possible. Presenting a stimulus led to a conjunctive neuron winning, and remaining active even after the stimulus was removed. However because this neuron's output synapses were not re-mapped to the encoded feature pattern, the corresponding feature neurons did not remain active during the delay. At recall, although the corresponding conjunctive neuron was activated, it was unable to re-activate the corresponding features by pattern completion (**Fig.S10B&C**). Thus WM recall requires plasticity in the readout of the conjunctive neurons.

Simulation 19: Feature-to-feature plasticity without conjunctive neurons

In this version of the model, we removed the conjunctive neurons. Instead we asked whether Hebbian plasticity in direct feature-to-feature synapses could perform the same tasks. The learning rules were replaced by:

$$\mathbf{W} \leftarrow \sigma\left(\mathbf{W} + \gamma(\mathbf{f}-\beta)(\mathbf{f}-\beta)^T - \mathbf{I}\right)$$

Here **W** is the flexible contribution to the feature-to-feature weights, represented by a 12 x 12 matrix, initialized randomly uniformly in the range [0,1], and γ is the learning rate for all the feature-to-feature synapses. The $-\mathbf{I}$ ensures the diagonals are zero, i.e. there is no learning for autapses. These flexible weight contributions **W** act in addition to the fixed lateral inhibitory weights between features in the same dimension, as described in the primary model:

$$\mathbf{f} \leftarrow \sigma\left(\beta + (\alpha_4 \mathbf{W}^{ff} + \alpha_5 \mathbf{I} + \alpha_6 \mathbf{W}) \cdot (\mathbf{f}-\beta)\right)$$

where $\mathbf{W}^{ff}$ is the static inhibition within feature dimensions, and **W** is the rapidly changing component of the weights. We adjusted the parameters of the model to permit sustained activity in a set of co-active feature neurons, giving: inhibition within dimensions $\alpha_4 = -0.07$, decay of feature activity $\alpha_5 = 0.9$, gain of inter-feature weights $\alpha_6 = 0.1$, and stimulus strength was increased to 3. As with our conjunctive neuron model, this network was optimised to produce similar performance to humans on the basic task. The model was able to produce basic set-size and serial position curves (**Fig.S10D**). It captured the recency but not the primacy effect. The effect of delay was tested, just as in Simulation 4 and no interaction with set size was observed (**Fig.S10E**). The incidental retro-cueing task, conducted as in **Simulation 6**, demonstrated a cueing effect on accuracy (**Fig.S10F**). Testing the effect of shorter encoding durations, just as in **Simulation 3**, demonstrated no change in initial encoding rate as a function of the number of items – however the asymptote strongly depended on the number of items encoded (**Fig.S10G**).

With long encoding durations, earlier items in the sequence were recalled poorly, indicating that the feature-to-feature synapses of earlier items were being overwritten.

Simulation 20: Synaptic decay time must be at least minutes

This simulation tests how the model performs when synaptic weights decay over time. Note that in the primary model, weights remain unless they are altered by ongoing plasticity. To examine how sensitive the model is to the timecourse of decay of plasticity, we replaced our plasticity rule with:

$$\Delta\mathbf{W}^{cf} = \gamma(\mathbf{c}-\beta)(\mathbf{f}-\beta)^T - \kappa\mathbf{W}^{cf}$$

$$\Delta\mathbf{W}^{fc} = \gamma(\mathbf{f}-\beta)(\mathbf{c}-\beta)^T - \kappa\mathbf{W}^{fc}$$

This meant that the learned synaptic weights decayed towards zero over time. If the constant kappa is zero, then this is identical to the primary model. We set the decay to have time constants ranging from 1/3 to 300 times the delay duration (**Fig.S11**). When the time constant corresponds to 10 times the delay period or longer, performance is no different (**Fig.S11A**). If the time constant falls below 3 delay periods, an extreme recency effect is observed and the model fails (**Fig.S11B**). This indicates that although the original rules had no synaptic decay in line with long-term plasticity, a shorter-term Hebbian plasticity such as that seen after a short weak tetanic burst, would suffice for working memory.

Simulation 21: Inverted neural representation of unattended items

It was noted that decoding for items that are not in the focus of attention remains above chance. However unattended representations should be suppressed by lateral inhibition – both at the level of feature neurons encoding features of the attended item, and also at the level of objects, by lateral inhibition between conjunctive neurons such that the unattended object representations are

suppressed. To examine the decodability in more detail, we used a 'cross-decoding' approach where we asked how the representation of an item it its attended state compared with its representation in the unattended state. Accordingly, we trained a linear classifier to predict which item was presented, training on trials where the item was in the focus of attention and testing it on trials in which it was unattended, and vice versa.

First we used the activity from the simulation in Fig.3 (Simulation 6: incidental retrocue). 400 simulated trials for each condition (cue-item 1 or 2) were divided into 2 sets of 200 trials, one group for training, and one for testing. A 4-way classifier decoded which of the possible orientations was presented, separately for item 1 and item 2, as a function of time in the trial (**Fig.S12A**). We compared decoding performance for within-condition decoding, i.e. when the same item was in the focus of attention, with performance for between-condition cross-decoding (**Fig.S12B**). After the incidental retrocue, which placed one item in the focus of attention, the classifier showed below-chance accuracy for cross-decoding. This demonstrates that the representation of the unattended item is 'inverted' relative to the focused item in memory. For comparison between conditions, the decoding accuracy in the delay period is re-plotted in **Fig.S12C**.

However we note that there is above chance decoding for item 2 even before item 2 is presented. This is because if a feature was in object 1, it could not occur in object 2. To get round this issue, we simulated a task in which the first item encoded in memory could take on one of three possible attributes, whereas the second item in memory was always fixed on that dimension, at the value that was never used for the first item (van Loon et al., 2018). In that study, this dimension was the 'stimulus category' – four categories were used, with one of the two stimuli

always a fixed category, whereas the other stimulus was from one of three other possible categories. To simulate this, the first item encoded had one of 3 possible orientation features, and the second item had a fixed orientation. Both objects also had a random colour and orientation. An incidental retrocue was presented (exactly as in simulation 6), to either shift attention to the first item, or to keep the second item in the focus of attention. We decoded at each timepoint which of the 3 possible orientations was presented for object 1. As above, this was performed either within-condition (**Fig.S12D**) or across-conditions (**Fig.S12E)**, to look for inversion of representation for focused vs unfocused items. Again we found below-chance cross-decoding after the cue, indicating that the representation of focused and unfocused items were inverted. The design of this study demonstrates that the inverse encoding cannot be due to generally inhibiting all feature neurons that are not in the attended item. Rather, there must be *selective* inhibition of the feature neurons of the unattended memory item (relative to items that were not present in the current trial).

In our model this arises because of inhibition between conjunctive neurons. Three out of four conjunctive neurons are inhibited, and each of these causes inhibition of their associated features. However the conjunctive-to-feature synapses are strongest for the item that was seen on the current trial, because of time and retroactive interference (as per **Fig.2D** and **2H**). In other words, the inhibited conjunctive neuron with the strongest interaction with the feature units corresponds to the unattended memory item. As a result, the features corresponding to the unattended item are selectively inhibited, even compared to features that were not presented on the current trial.

Simulation 22: Increasing the number of conjunctive neurons

One reason for a limited capacity—that could drive interference—is the small number of conjunctive neurons. If only four conjunctive neurons are present, then only four “slots” are

available for binding features. However, we can increase the number of conjunctive neurons without increasing the capacity, simply by tuning the mutual inhibition between these neurons. In fact, the key property of the network that limits capacity in this way, is that there are only four orthogonal configurations of the active conjunctive neurons—in particular, a quarter of neurons are maximally active at once. So for a network of 8 conjunctive neurons, we can reduce the inhibition such that two conjunctive neurons are active at any one time, rather than one, to create the same capacity limit as before. Thus we used 8 conjunctive neurons, and reduced α_1 from 0.28 to 0.10. The results are shown in **Fig.S14**.

Simulation 23: Repeated features on the irrelevant dimension

A unique aspect of the present model is that it makes novel predictions about the effects of feature repetition. In previous simulations, all objects differed from each other on all three dimensions. Here we presented three objects, but allowed objects to sometimes share features on the irrelevant dimension. We considered repetitions where objects 1 & 2 shared a feature, but object 3 was distinct on the third dimension, and similarly for repetitions in objects 2 & 3, 1 & 3, and the situation where the irrelevant dimension had the same feature for all three objects. Simulations proceeded otherwise identically to the standard 3-item condition. Accuracy is reported when each of the 3 items was probed, in **Fig S16**.

Simulation 24: Retrieval of whole objects vs. individual features

Here we investigated whether errors are correlated between features, when recalling objects. Simulation was exactly as per simulation 1. Trials were then divided up based on the final feature-unit activity for the irrelevant dimension (**Fig.S17**). Each trial was marked as "irrelevant dimension correct" if the feature activity at the end of the trial was greatest for the irrelevant

feature corresponding to the probed item, and incorrect otherwise. Accuracy was calculated as usual, separately for these two groups of trials.

There are two contributions to errors. The first component is driven by incorrect re-activation, where the probe activates the incorrect conjunction neuron. In this case, the incorrect features will be activated, on both the relevant and irrelevant dimensions, at the same time. The second component of error is driven by incorrect synaptic weights at the time of recall. This arises primarily during the encoding phase – either because of inadequate activation of a new conjunction neuron when the object was presented, or because of subsequently-encoded memory items partially overwriting the synaptic weights of the object. Either way, this results in the feature of one dimension being recalled correctly, while the other dimension is recalled incorrectly. Accordingly, correlated errors observed most strongly for items that must be re-activated from a silent state, whereas all items show a degree of uncorrelated errors.

Minimal algorithm to reproduce main results

```
α ← [ -0.28, 1.03, 0.05,    -0.28, 0.75, 0.05  ]
β ← 0.175
γ ← 0.02
Wff ← α5 eye(12)  + α4 [
    1 1 1 1 0 0 0 0 0 0 0 0
    1 1 1 1 0 0 0 0 0 0 0 0
    1 1 1 1 0 0 0 0 0 0 0 0
    1 1 1 1 0 0 0 0 0 0 0 0
    0 0 0 0 1 1 1 1 0 0 0 0
    0 0 0 0 1 1 1 1 0 0 0 0
    0 0 0 0 1 1 1 1 0 0 0 0
    0 0 0 0 1 1 1 1 0 0 0 0
    0 0 0 0 0 0 0 0 1 1 1 1
    0 0 0 0 0 0 0 0 1 1 1 1
    0 0 0 0 0 0 0 0 1 1 1 1
    0 0 0 0 0 0 0 0 1 1 1 1
```

```
]
Wcc ← α2 eye(4) + α1 ones(4)

c ← [0 0 0 0]T
f ← [0 0 0 0  0 0 0 0  0 0 0 0]T
W ← rand(12,4)                    // as γ1 == γ2, Wfc == Wcf T
t ← 0
while t < stimulus.length {
      f ← β + Wff (f − β) + α3 W (c − β) + stimulust
      f ← max(0,min(1,f))
      c ← β + Wcc (c − β) + α6 WT (f − β) + 0.005 * randn(4,1)
      c ← max(0,min(1,c))
      Δ ← (f − β)(c − β)T
      W ← max(0,min(1, W + γ Δ))
}
choice ← findmax( f[4:8] )

stimulust ← {
    200 × [-1 -1 -1 -1  -1 -1 -1 -1  -1 -1 -1 -1]  // foreperiod
    120 × [+1 -1 -1 -1  +1 -1 -1 -1  +1 -1 -1 -1]  // obj1
     50 × [ 0  0  0  0   0  0  0  0   0  0  0  0]
    120 × [-1 +1 -1 -1  -1 +1 -1 -1  -1 +1 -1 -1]  // obj2
    300 × [ 0  0  0  0   0  0  0  0   0  0  0  0]  // delay
    120 × [+1 -1 -1 -1  -1 -1 -1 -1  -1 -1 -1 -1]  // probe
    220 × [ 0  0  0  0   0  0  0  0   0  0  0  0]
}
```

Supplementary Figures

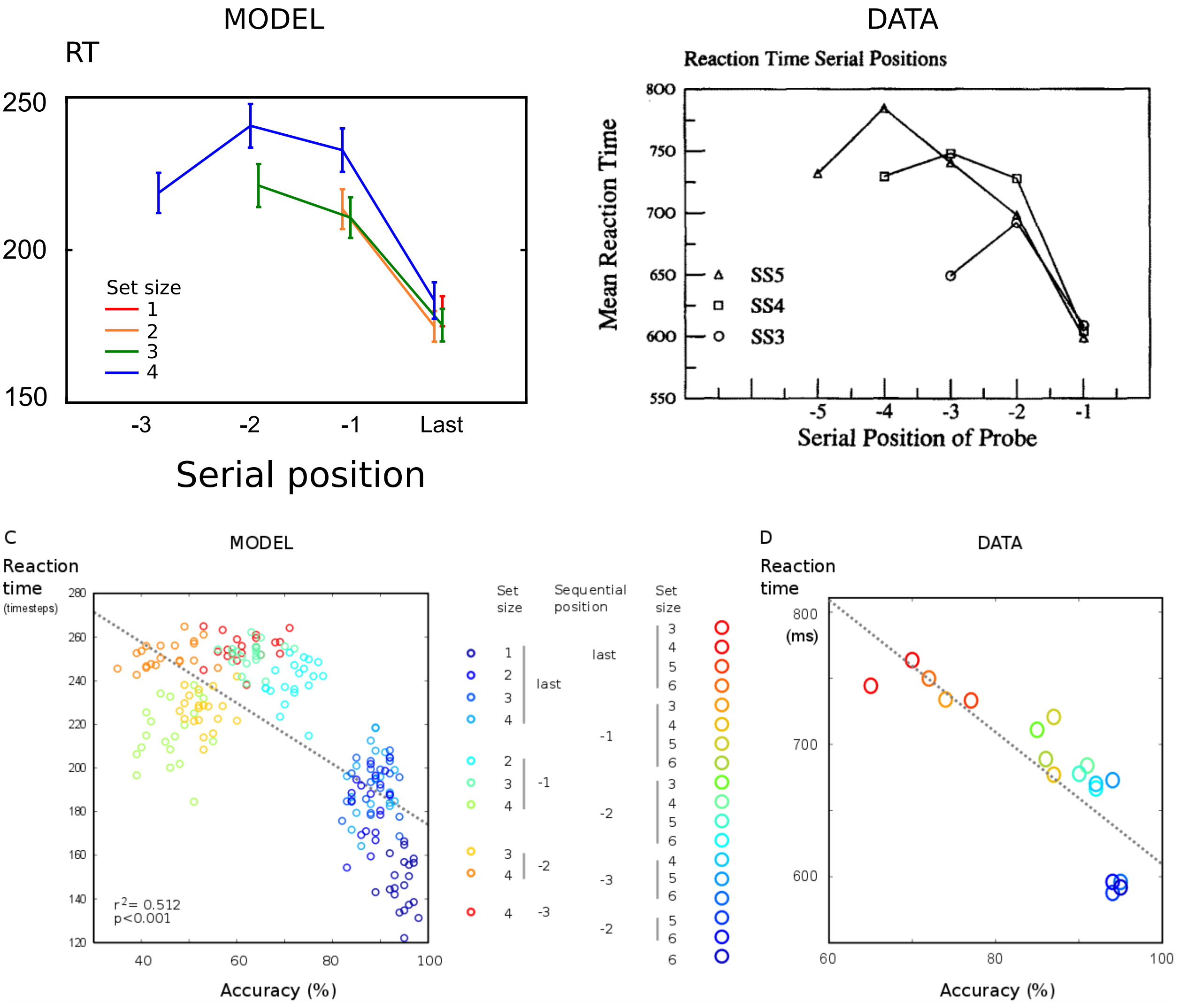

Figure S1: Reaction times (Simulation 7)

The time taken to recall an item can be indexed by how fast the winning feature rises after probe presentation. An inverse relation with accuracy is observed, in keeping with behavior, with items already in the focus of attention showing the fastest responses. (**Simulation 8** below)

A) Reaction times were extracted from trials of simulation 1, quantified as the time after the probe at which the winning feature reached 95% of its maximum value. The faster the attractor settled into a stable winning state, the shorter the reaction time. Each line represents a different set size, and the X-axis indicates serial position of the probed item within the memory set. Reaction times were approximately inversely related to accuracy on each condition.

B) This is consistent with race-models of WM recall (Pearson et al., 2014), and aligns with behavioural data (McElree, 2006; McElree and Dosher, 1989)(Figure adapted from McElree & Dosher 1989).

C) Correlation between accuracy and reaction times for the 10 conditions (4 set sizes, for each serial position). For each condition 20 subjects each with 100 trials were simulated. As accuracy increases, reaction times get shorter. The values are taken from the simulations shown in **Fig. 2E**

and **Fig. S1A**) Data re-plotted from pilot study in McElree and Dosher (1989), showing the mean RT and mean accuracy across participants.

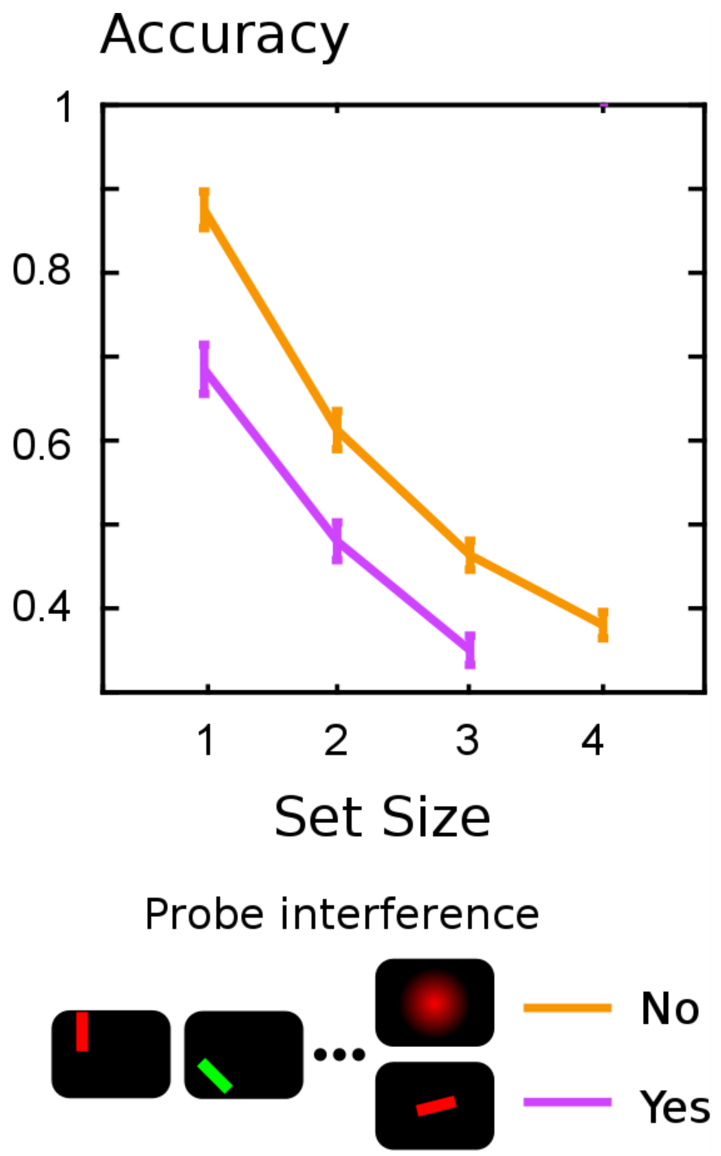

Figure S2: Novel prediction – Probe interference (Simulation 8)
The model predicts that the probe can itself interfere with recall if it contains irrelevant features. The irrelevant features compete with the probe feature, and reduce the probability of correct re-focusing of the item, in line with data (Tabi, Husain and Manohar, 2019).

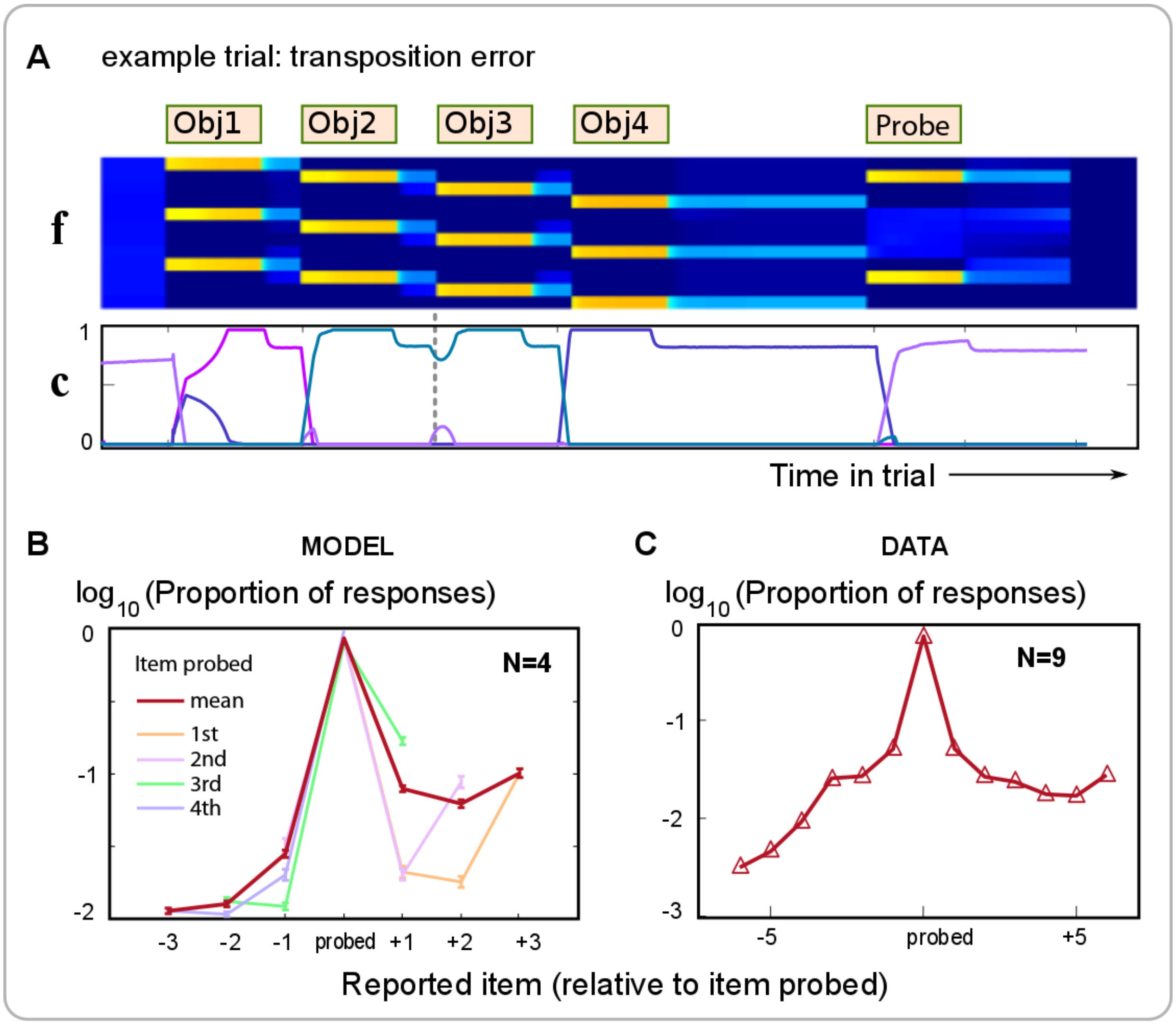

Figure S3: Errors are more likely to be sequentially neighbouring items (Simulation 5)
A Transposition errors (sequence positional errors, or swap errors) arise when features of a temporally adjacent item are instead reported. In this example trial, the conjunction neuron activated by object 2 remained active for object 3 (vertical dashed line), so when item 2 or 3 is probed, the incorrect response feature is sometimes activated.
B Items that were either just before ($x<0$), or just after ($x>0$), the probed item tended to be reported more often.
C A qualitatively similar pattern of errors is observed in a verbal WM task in which a list of words had to be recalled in order (adapted from Expt.3 of Farrell and Lewandowsky, 2004). A very similar falloff with inter-item distance is also seen in visuospatial memory using sequence change-detection (Smyth and Scholey, 1996), but their data did not permit distinguishing forward and backward transpositions.

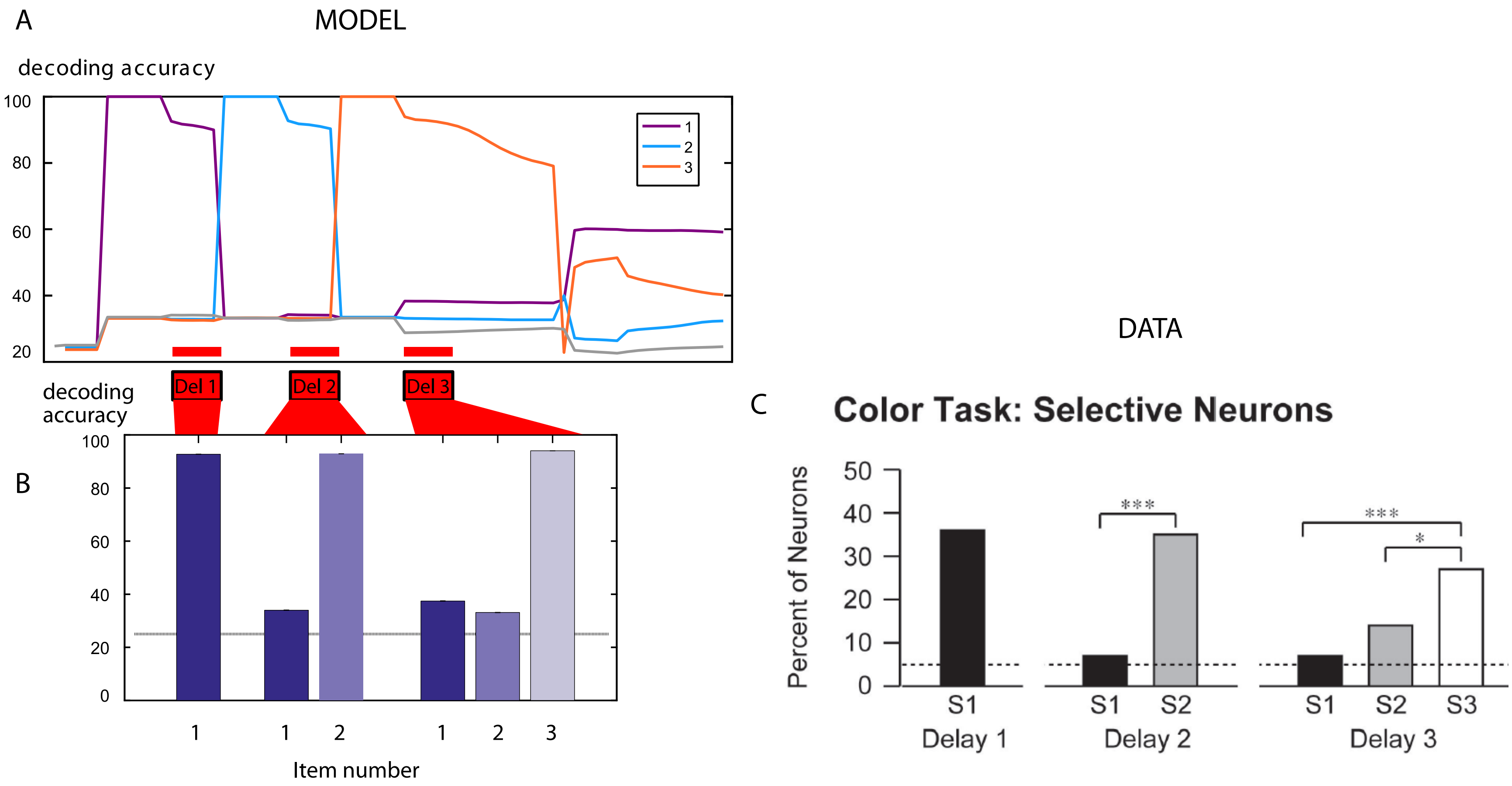
A
MODEL
decoding accuracy
100
80
60
40
20
1
2
3
Del 1
Del 2
Del 3
decoding accuracy
B
100
80
60
40
20
0
1
1
2
1
2
3
Item number
DATA
C
Color Task: Selective Neurons
Percent of Neurons
50
40
30
20
10
0
***
***
*
S1
Delay 1
S1
S2
Delay 2
S1
S2
S3
Delay 3

Figure S4: Decoding from feature units during the delay period (Simulation 11)
Feature-neuron activity during the delay period of the 3-item condition was examined. For each time point, the activity of the four color units was used to decode (across trials) the identity of each presented color. A linear classifier was trained on the activity in the feature units **f** on 50% of trials, and tested on the remaining trials. A) The decoder accuracy for each of the three items is shown, as a function of time. The three delay periods of interest, following the presentation of each item, are shown as pink bars below. B) Average decoder accuracy during each of the delays. During the first delay we could decode the identity of the first item's colour. During the second delay, we could decode the second items' color but only very weakly the first item's color. In the third delay we could decode the third item's color, but the other two colors were only weakly decodable. Dotted line is chance. The weak decoding of unattended items is analysed in more detail in **Fig.12**, Simulation 21. C) Data from (Konecky et al., 2017), showing decodability of only the most recent item presented during a monkey WM task. The neurons were in fact from the principal sulcus, and 35% of neurons recorded here were feature-selective, though the remaining neurons were not.

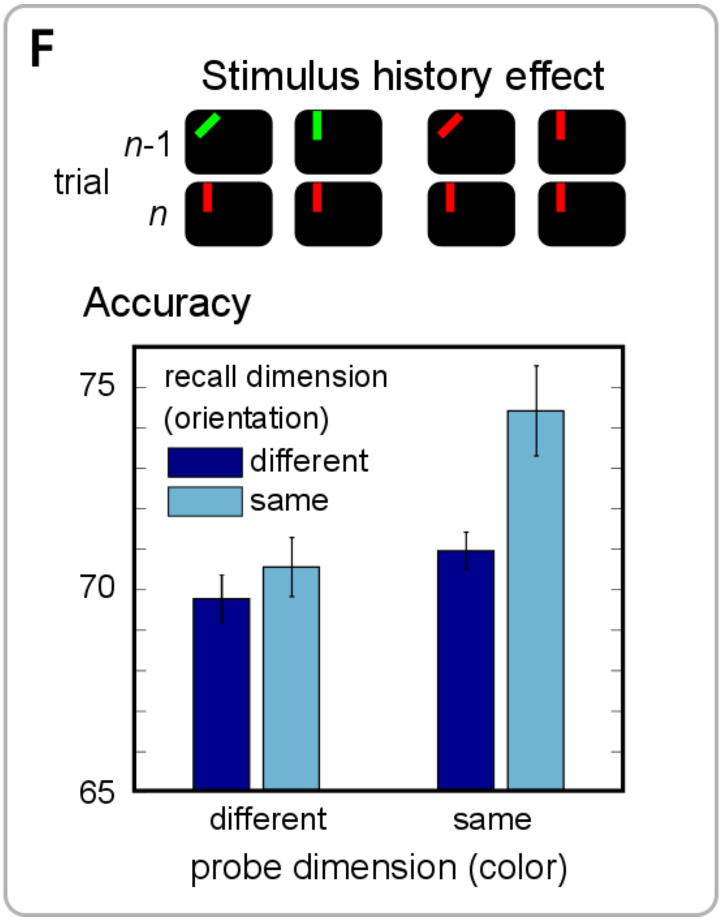

Figure S5: Novel prediction: Trial-to-trial conjunctions effect (Simulation 12)
Since conjunctive neuron selectivities rely upon synaptic traces from the trial history, we predict that the stimuli presented on the previous trial generate interference effects with the current trial. The model predicted that when the probed item's features were identical to those of the item probed on the previous trial, responses were more accurate.

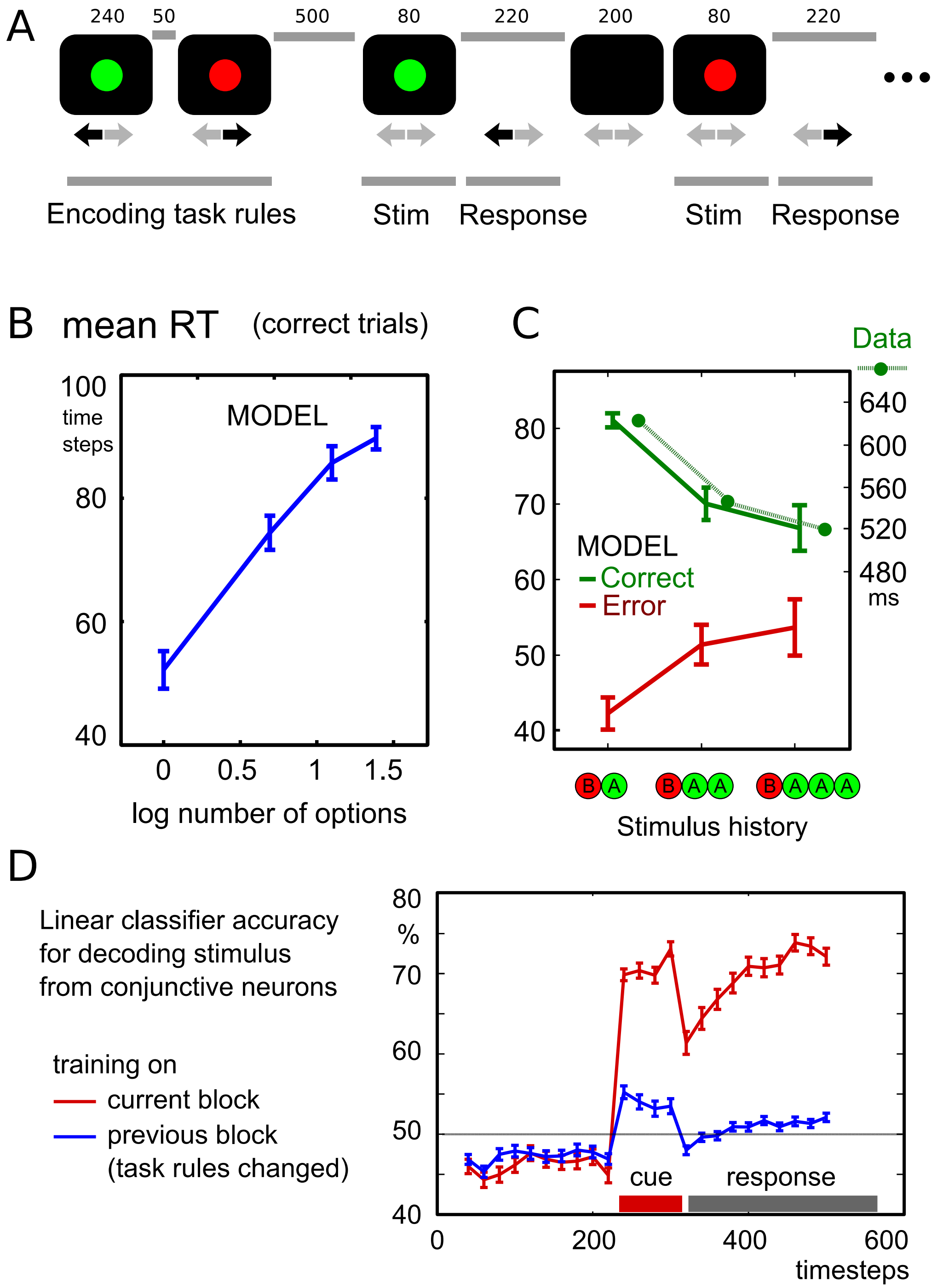

Figure S6: Acting on multiple task rules (Simulation 14)
Here we simulated a simple N-alternative choice task, where each colour indicates that a particular button should be pressed.
A) At the start of each block, a set of task rules was encoded. A single color and motor plan feature were activated simultaneously, indicating the rule for example “if green, press left”. After 1 to 4 rules were presented, a series of test trials followed. One of the colour features that was

presented previously was activated, corresponding to presenting a cue. The subsequent response unit activation was measured, to indicate the model's response. Reaction times were calculated as previously from the time of stimulus onset.
B) The RT increased with the logarithm of the number of rules encoded, according to Hick's law.
C) RTs were split according to stimulus repetition history. If the same stimulus was tested on the previous trial ('BAA'), or on the previous two trials ('BAAA'), then the RT on correct trials was faster than if the stimulus was different ('BA'), in keeping with data. Dotted line: RT on correct trials replotted from Expt 4 of (Schvaneveldt and Chase, 1969), in which one of 4 responses was selected after seeing one of 4 stimuli, according to an arbitrary stimulus-response mapping. The model also predicts errors will be faster than correct responses, with an inverted stimulus-repetition effect.
D) Unlike in the WM task, decoding is possible from conjunctive units, since the task set is maintained rather than overwritten on each trial. For each test trial, the stimulus identity was decoded using the other trials in the same block for training (red), or the trials in the previous block (blue). Decoding was possible within the block, because a consistent conjunction unit pattern – representing the task rule corresponding to the current stimulus – was activated.

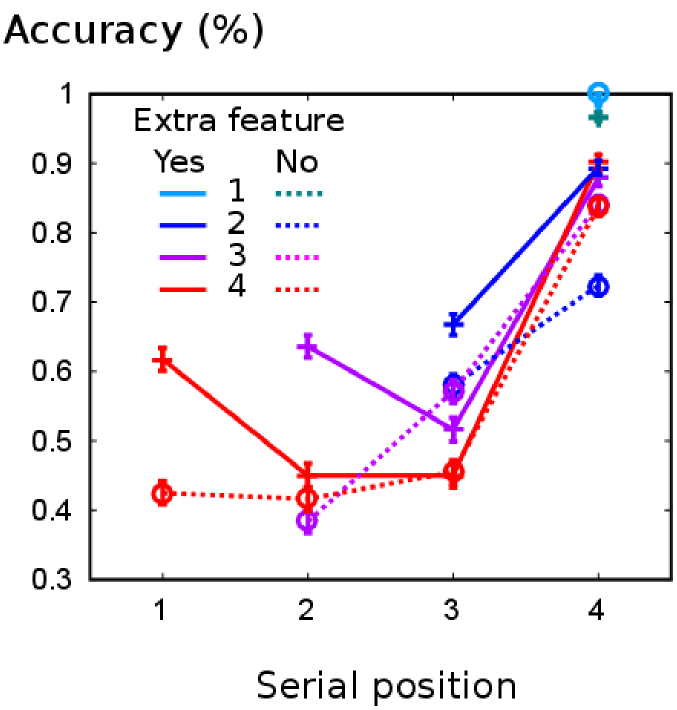

Fig. S7: Novel prediction: Benefit with an extra feature dimension (Simulation 15)
Previous simulations had three features per object (colour, orientation and location), of which only two were task-relevant. The third, irrelevant, feature was distinct for each object. Removing the task-irrelevant feature (e.g., presenting sequential items all at one location, rather than at different locations) worsens model performance, in particular by reducing primacy and recency benefits.

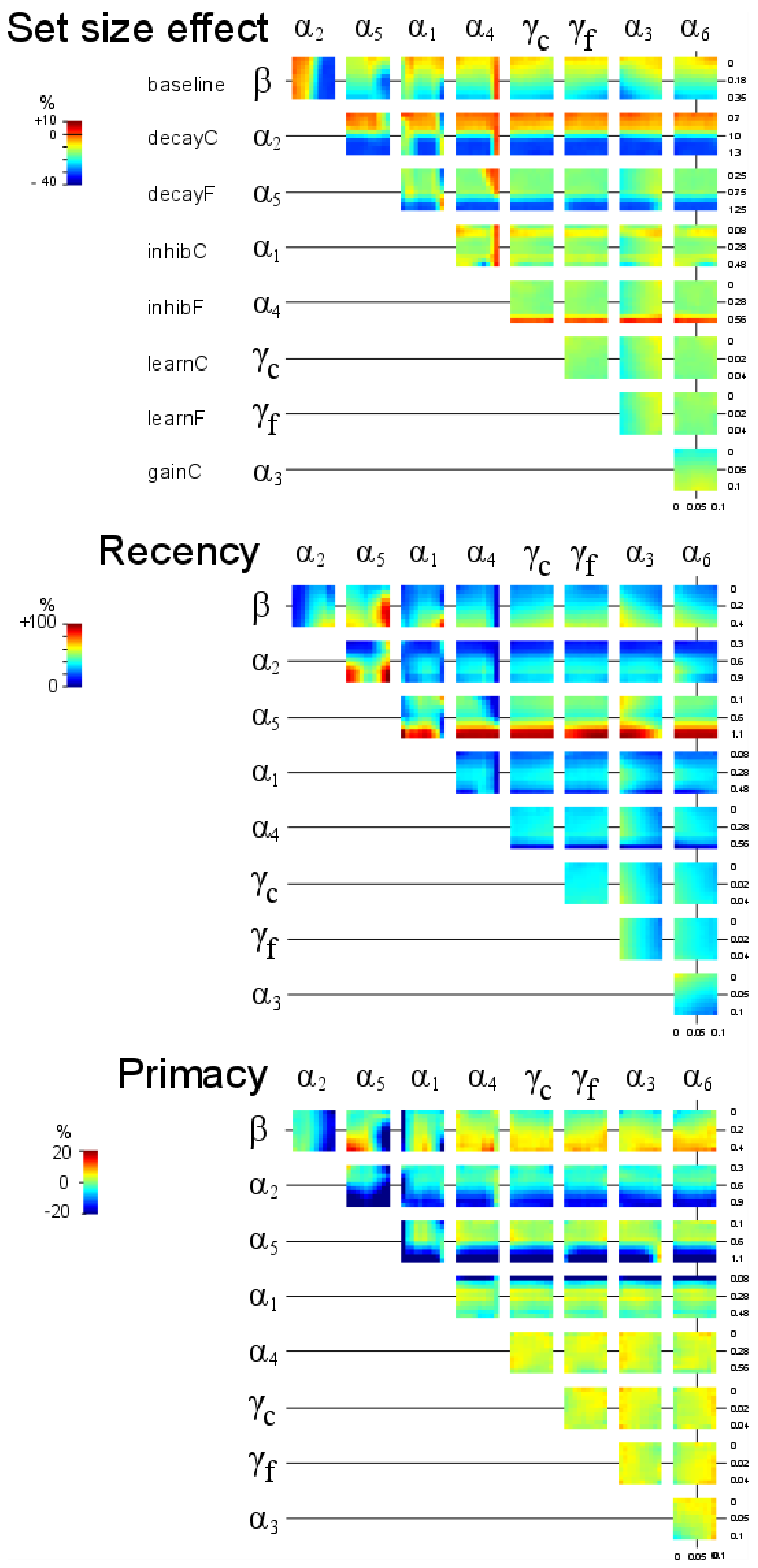
Set size effect
baseline
decayC
decayF
inhibC
inhibF
learnC
learnF
gainC
Recency
Primacy
%
+10
0
- 40
+100
20
-20

Fig.S8: Influence of model parameters upon central behavioral effects (Simulation 16)
We examined the effect of varying 9 free parameters in the model, varying two of them at a time. The parameter values used in the main paper lie at the centre of each 10 x 10 grid, and the figure represents all possible pairs of free parameters. For each parameter combination, we quantified the set size effect (reduction in accuracy as set size increases), primacy effect (difference in accuracy between final and penultimate items in sequence) and recency effect (difference in accuracy between first and second items in sequence. Warm pixels indicate larger effects.

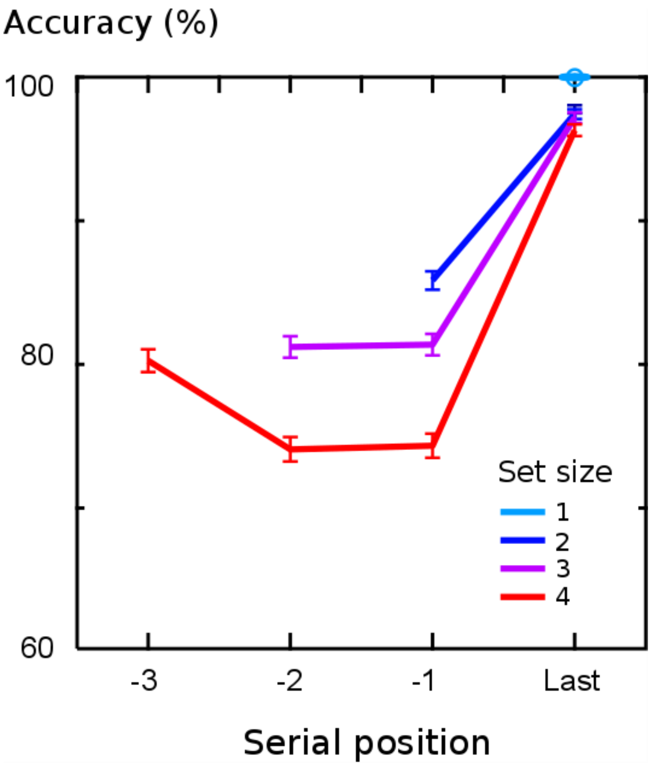

Fig.S9: Basic results for ‘high-accuracy’ parameter regime (used for simulations 3 to 5)
To prevent floor effects when simulating encoding and delay effects, we required a higher initial performance level, and adjusted the parameters accordingly. This figure demonstrates the equivalent of Figure 2D, using this new set of parameters. Performance is overall higher but demonstrates qualitatively similar effects.

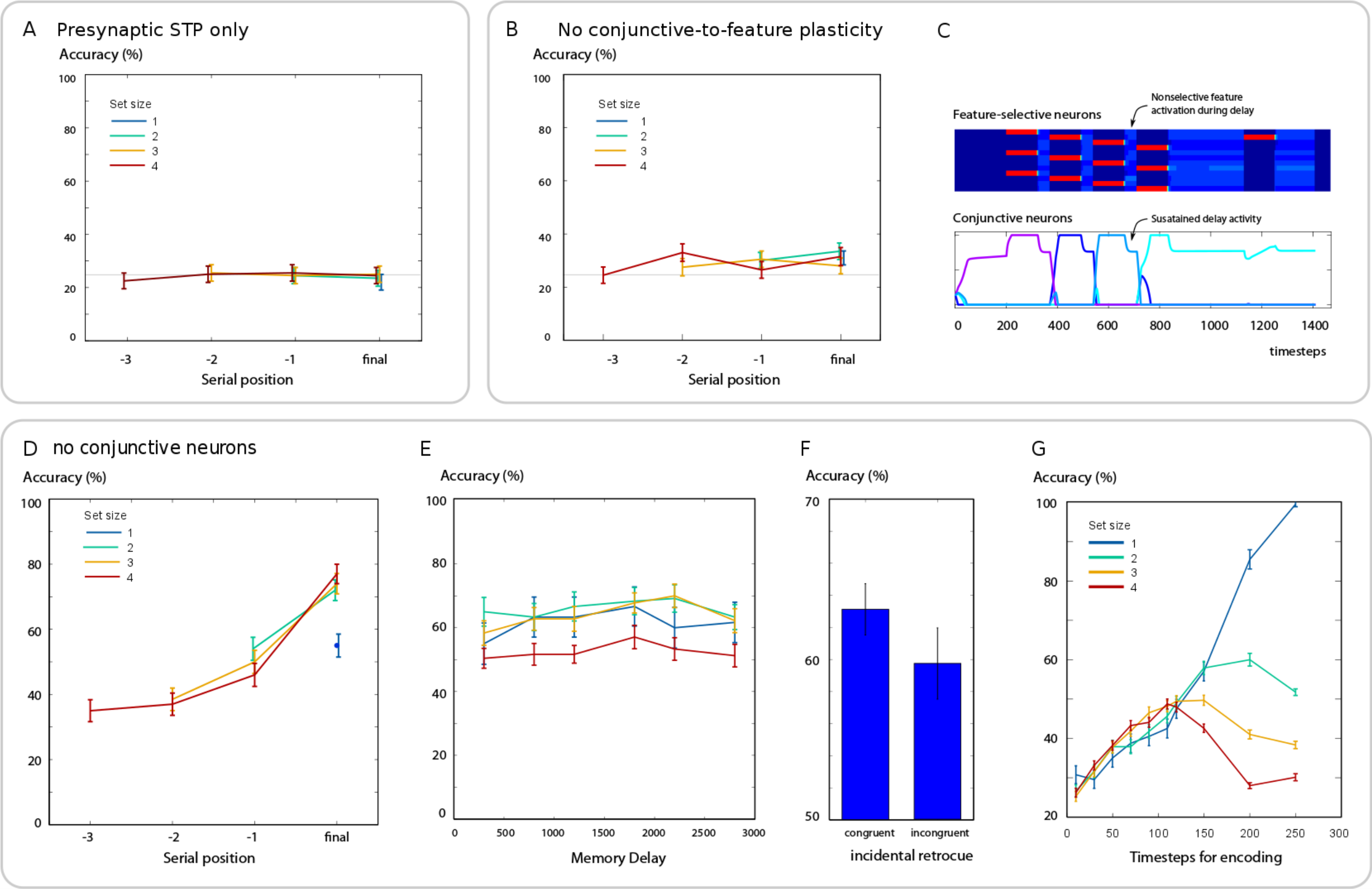

Fig.S10: Failure of simplified versions of the model (Simulations 17, 18, 19)
These simulations aimed to demonstrate if the model could operate with fewer assumptions. A) Hebbian plasticity was replaced by presynaptic short-term potentiation, which is insensitive to the activity in postsynaptic neurons. The model was unable to produce sustained activity, and recall is at chance. B&C) Plasticity of the synapses from the conjunctive neurons to the feature-selective neurons was removed. In this situation, encoding causes on conjunctive neuron to win out, and synapses from the active features to this conjunctive neuron are strengthened. This is sufficient to drive sustained activity in the conjunctive neurons. But when the input is removed, it is not retained in an active state, because the active conjunctive neuron does not selectively drive the same features. Thus there can be no recall by pattern completion, and performance is at chance.

D) The conjunctive neurons were removed, and bidirectional Hebbian plasticity was implemented at the feature-to-feature synapses. With some fine tuning, it was possible to produce sustained activity through just these feature-to-feature synapses. This also permitted recall of cued items by pattern completion. Moreover, parameters could be adjusted so that the network matched some aspects of human performance. In particular a set size and serial position effect could be obtained. E) However, there was no interference as a function of the memory delay – i.e. storing additional items did not cause faster degradation in the delay. F) This network did produce retro-cueing effects: activating one feature shifted the persistent activity to the object

associated with this feature. G) Encoding failed to show an interference effect, in that the initial rate of encoding was independent of set size. Additionally, when encoding was too long, the last item to be encoded overwrote the feature-to-feature synapses of previously stored items.

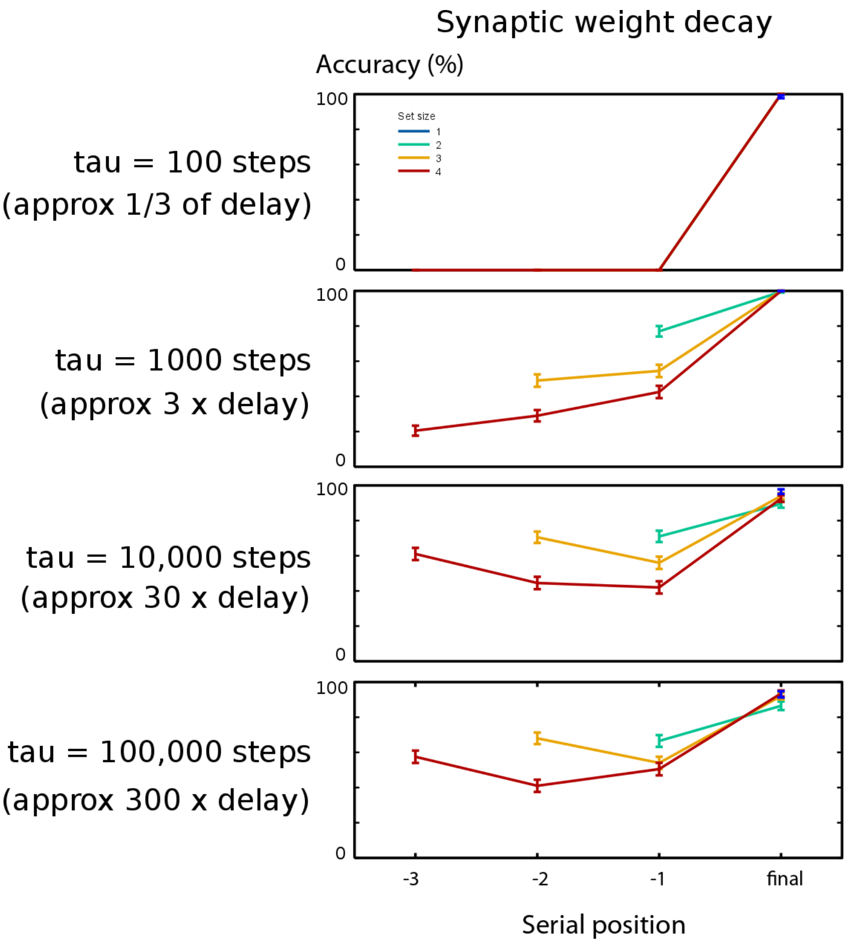

Fig.S11: Allowing decay of synaptic weights (Simulation 20)
The basic simulation was run with synaptic weight decay over time, with an exponential time constant tau. Set size and serial order effects remained intact as long as the time constant was an order of magnitude longer than the delay duration.

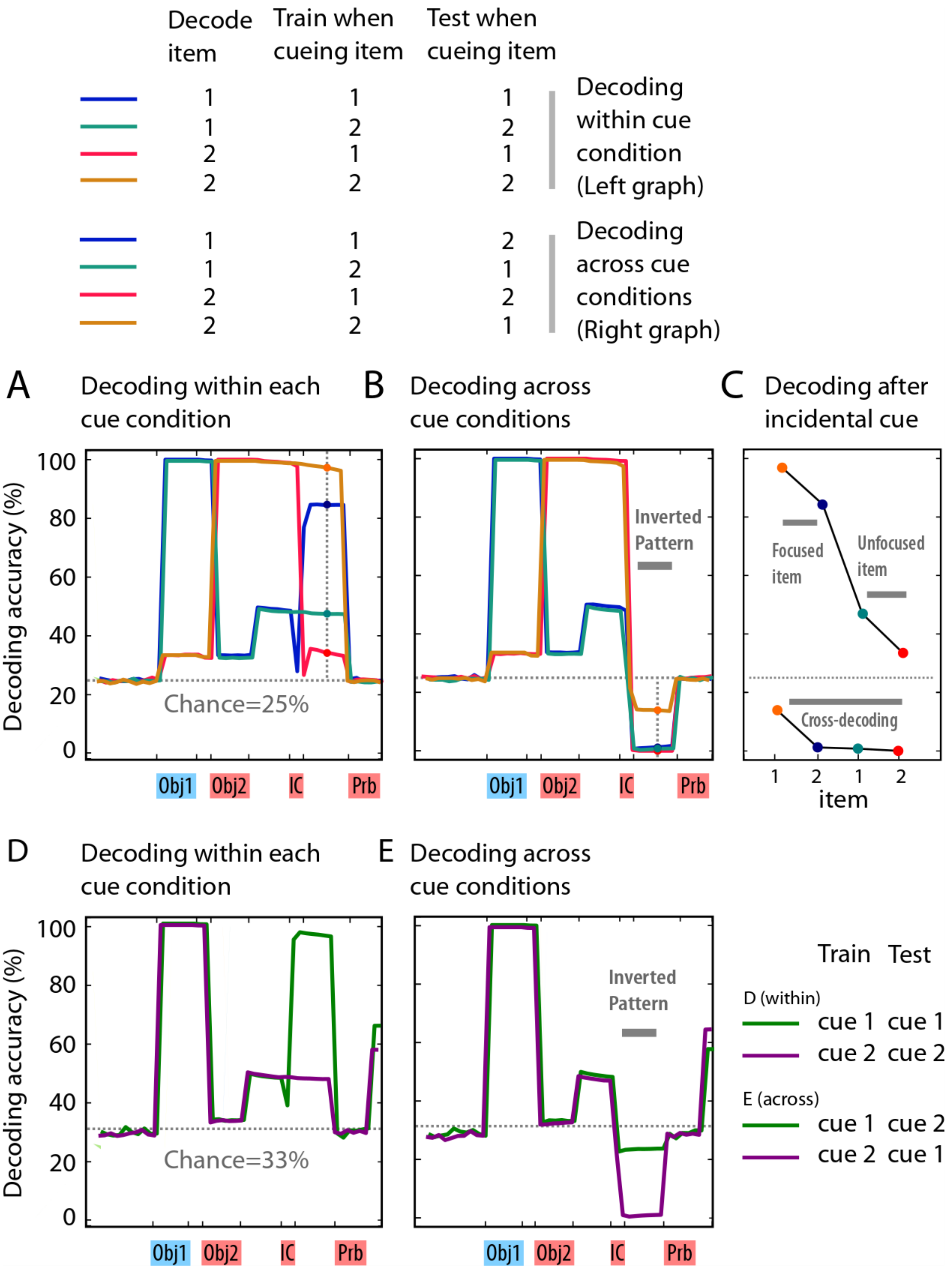
Decode item
Train when cueing item
Test when cueing item
1 1 1
1 2 2
2 1 1
2 2 2
Decoding within cue condition (Left graph)
1 1 2
1 2 1
2 1 2
2 2 1
Decoding across cue conditions (Right graph)
A
Decoding within each cue condition
B
Decoding across cue conditions
C
Decoding after incidental cue
Decoding accuracy (%)
Chance=25%
Obj1
Obj2
IC
Prb
Inverted Pattern
Focused item
Unfocused item
Cross-decoding
item
D
Decoding within each cue condition
E
Decoding across cue conditions
Chance=33%
Train Test
D (within)
cue 1 cue 1
cue 2 cue 2
E (across)
cue 1 cue 2
cue 2 cue 1

Fig.S12: Cross-decoding reveals inverse coding of unattended items (Simulation 21)

Here we asks whether the pattern associated with a stimulus during the memory interval is different when the item is attended vs unattended. To examine the nature of the representation of the unattended item, we compared decodability of the attended and unattended items, either when training and testing within the same retro-cueing condition, vs. when training and testing when the other item was in the focus of attention.

A&B) We used the incidental retrocueing task (**Fig.3A**, Simulation 6), in which two items were presented sequentially, then during the delay, one of the two items was brought back into the focus of attention by cueing its colour feature only. We decoded items using a linear classifier on the feature units, which predicted which of the four possible orientations was presented in a given memory item. Traces show decoding accuracy for items 1 (cool colours) or 2 (warm colours) from trials where either item 1 or 2 was incidentally cued (IC). **A**) During the delay after the IC, the cued item is decoded well, and the uncued item less well – though still above chance.

B) Cross-decoding: When training on the item in the focus of attention, and decoding that same item when it is unfocused, classification is below chance, indicating an 'inversion' of the representation. This is because the pattern of neurons active when the feature is attended, is suppressed below baseline when it is unattended. **C**) shows decoding performance in the post-cue delay for each condition, taken at the vertical dashed line in **A&B**.

D) The analysis of the incidental cueing task is complicated because when a feature is present in one item, it cannot be present in the second item, so the items are not independent. To overcome this we simulated the design of van Loon et al. (2018) in which only the first item had variation along the feature of interest for decoding, having one of 3 possible feature values. The second item was fixed on this dimension. We decoded the identity of item 1 only. As expected, when the second object is shown, decoding was reduced, but above chance in the first part of the delay, indicating that residual information was present about this item. Cueing the first object restored decodability (green line after IC).

E) The critical comparison is cross-decoding of item 1's identity when it is attended vs. unattended. Decoding is below chance after the IC, indicating that when the item is unattended, it is encoded in an inverted pattern relative to when it is attended.

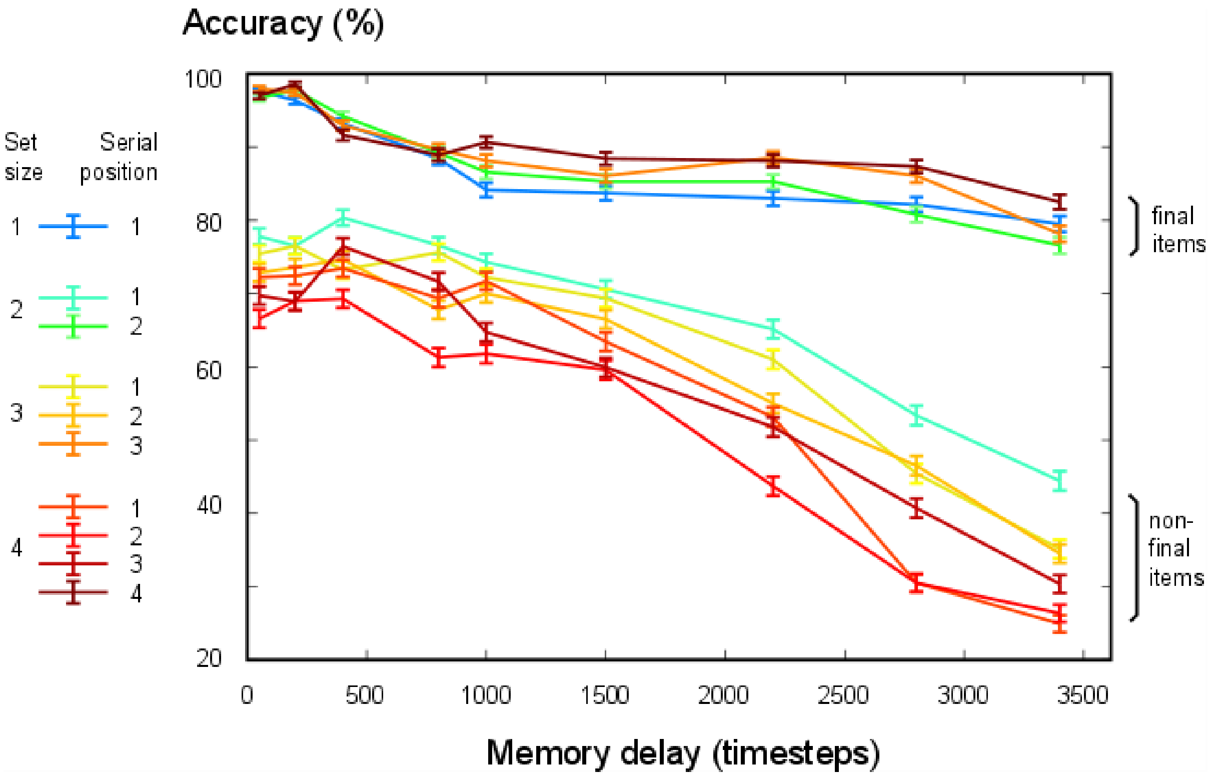

Fig.S13: Decay effect during the delay period split by serial position
Here we plot the data shown in **Fig.2H** (Simulation 4) as a function of serial position as well as set size. This shows that the initial decrease is driven by the most recently presented item undergoing immediate decay. Note that this effect is not seen in the data. The last item is robust to decay, whereas the synaptic traces of non-final items decrease in strength because of the ongoing Hebbian learning rule.

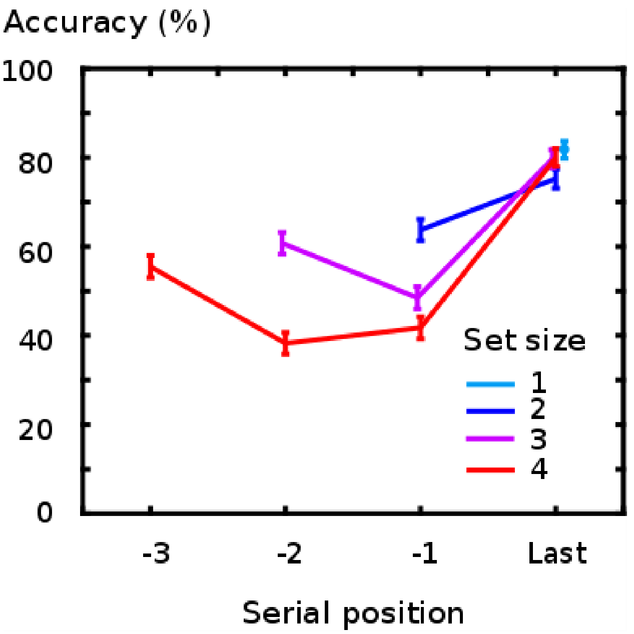

Fig. S14: Increasing the number of conjunctive units to 8 (Simulation 22)
The capacity limit of the model is not determined solely by the number of conjunctive neurons being 4. By adjusting the level of inhibition, the number of conjunctive neurons can be increased while maintaining the capacity limit at 4 items. This occurs when two conjunctive neurons become active at a time. When more than four items are presented, subsequent items must re-activate conjunctive neurons that were previously used to bind previous items – resulting in retroactive interference.

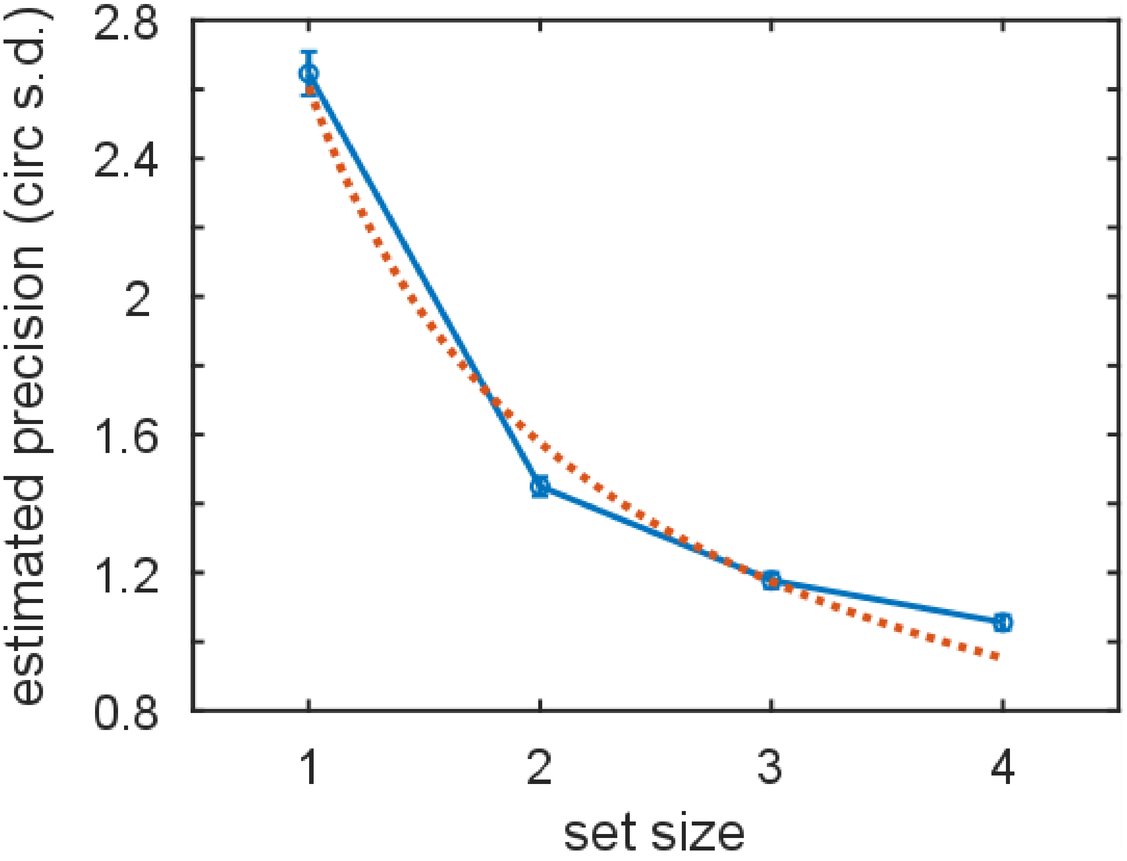

Fig. S15: Estimated precision follows power-law with set size (Simulation 1)
Resource models of WM have suggested that the precision with which a feature is remembered falls as a power law with respect to the number of items that must be stored. Our simple model has no proximity structure between features, and so does not explicitly predict the precision of WM. However if we assume that reporting the wrong item indicates items that are equally spaced around a circular feature space, we can estimate the effective precision that would be obtained for this error rate. To do this, the circular standard deviation for each set size was calculated while randomising each non-target feature to a non-target “angle”: $-\pi/2$, $+\pi/2$, and π (points: mean circular standard deviation, and bars: s.e.m. of this value across 100 subsamples of the simulation). This surrogate precision was fitted to the power law (dotted curve), giving an estimated exponent of 0.76 (empirical value ~0.74; Bays & Husain 2008)

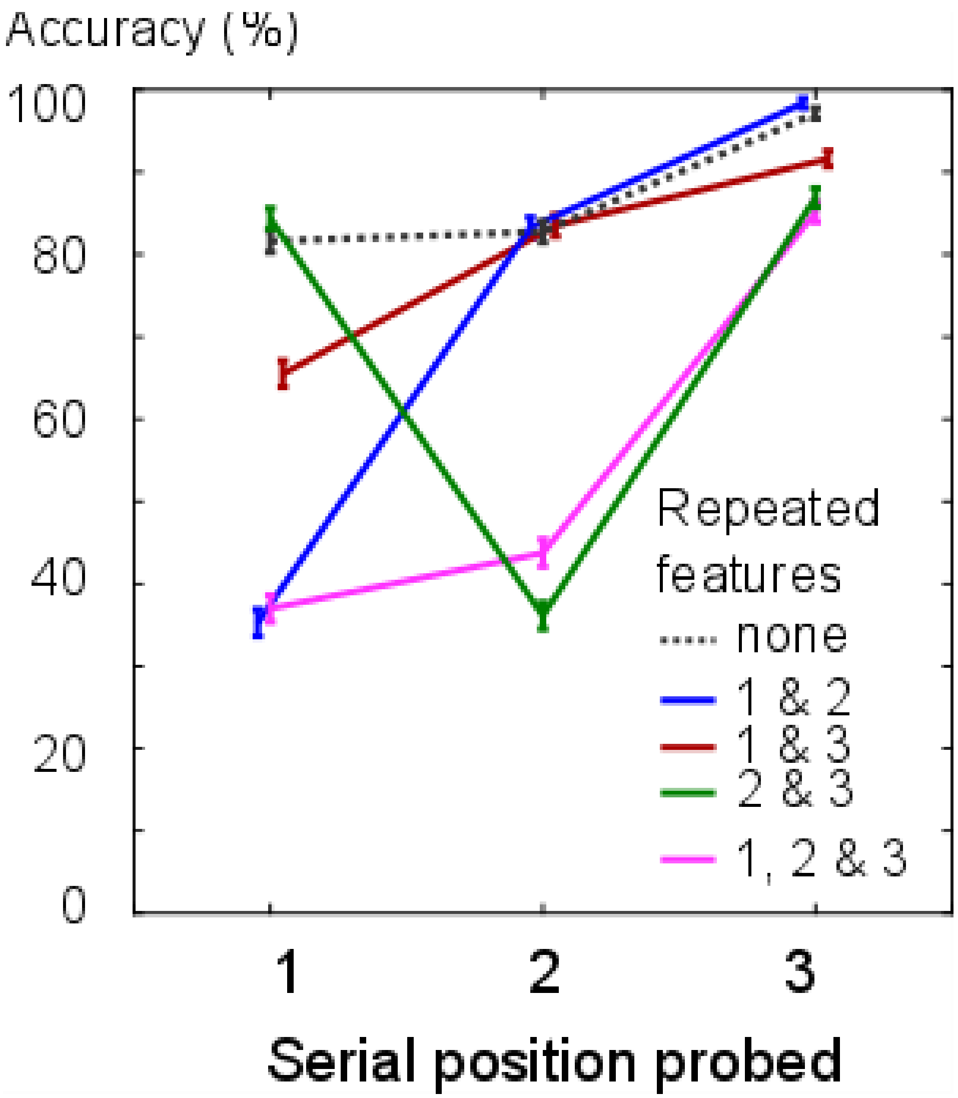

Fig. S16: Novel Prediction: Effects of repetition of irrelevant features across items (Simulation 23)

When irrelevant features are present, they act to facilitate separation between items. Here, three items were presented, which were different on the two relevant dimensions, but the irrelevant dimension sometimes had the same feature for two of the items in the sequence. E.g, “repeated features 1&2” means that items 1 and 2 shared the same feature on the irrelevant dimension, whereas the third item had a different feature. Simulations showed that, when the irrelevant feature is identical across some of the items in the sequence, memory accuracy is reduced for those items. However, this distinctness effect interacts with recency: the last item in the sequence is robust to these effects.

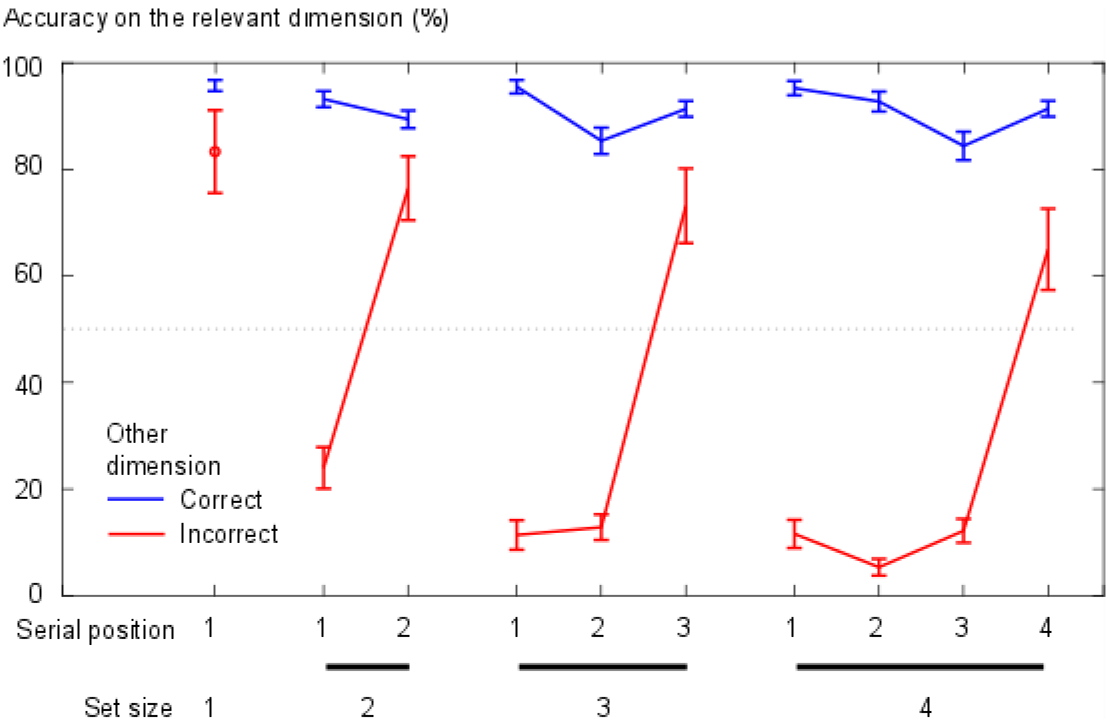

Fig. S17: Novel Prediction: Correlated and uncorrelated recall of two features of the same objects (Simulation 24)

Empirical studies have asked participants to report two features of the same object, and find surprisingly low correlations between the two features. These have been considered as evidence against the “object-based” account of memory failure. We therefore examined re-activation of features on the irrelevant dimension (i.e. in this case, orientation), as well as the relevant one (location), after the probe (colour). Trials were split according to whether the features on the irrelevant dimension correctly reflected the probed item at the end of the trial – i.e., whether the irrelevant dimension would have been reported accurately. Blue lines indicate trials where the irrelevant feature was correct, red lines indicate trials where the incorrect feature was active.

In both cases, accurate orientation reports occurred even when irrelevant feature was erroneously reported, and orientation errors were made even when the irrelevant feature was correctly reported. The correlation between errors on the two dimensions visualized by the distance between the red and blue line. Strikingly, when the probed item was not the last item in the sequence, errors in the two dimensions were relatively uncorrelated, whereas for the other items, errors were more strongly correlated.

Table S1: Empirical findings explained by the model

Phenomenon explained	Mechanism in model	Refs
**Set size** reduces recall accuracy	Competition between conjunction units → interference	(Zhang and Luck, 2008)
**Primacy** – first item benefit	No competition with previous items at encoding	(Baddeley, 1996)
**Recency** – last item benefit	Features retained in active state; no retrograde interference	(Gorgoraptis et al., 2011)
Only last item **encoded actively** in firing rates	Subsequent items capture focus of attention	(Konecky et al., 2017)
**Shifting attention** to item improves recall	Re-activation by pattern completion focuses attention on item by sustained activity. Subsequently probing that item is faster and more accurate, as it is already active.	(Myers et al., 2017; Souza et al., 2016; Zokaei et al., 2014b)
**Additional features** in an object remembered with low cost	During encoding, synaptic weights increase in parallel to all concurrently active features.	(Allen et al., 2006; Luck and Vogel, 1997; Sala and Courtney, 2007)
**Frontoparietal activation** during WM maintenance	Conjunctive and feature neuron firing required for focus of attention and thus for encoding	(Rowe et al., 2000; Sakai et al., 2002)
**Frontoparietal connectivity** is signature of shifting attention	Synapses between conjunctive and feature units are bidirectionally activated to drive persistent activity	(Heinen et al., 2017; Scolari et al., 2015; Szczepanski et al., 2013)
**Synchrony** between posterior and frontal regions during WM	Reciprocal excitation between conjunctive and feature neurons crucial for stable attractor of focus of attention.	(Fries et al., 2001; Gregoriou et al., 2009)
Fine-grained decoding from PFC during WM is elusive	Conjunctive neurons encode information flexibly across trials, with selectivity depending on recent history	(Cogan et al., 2017; Harrison and Tong, 2009; Lara and Wallis, 2014; Sprague et al., 2016)
**Inversion** of neural encoding when items are unattended	Lateral inhibition between conjunctive neurons and between feature neurons suppresses feature of the unattended item below baseline.	(Rademaker et al., 2018; van Loon et al., 2018; Yu and Postle, 2018)
Working memory operates as an **attentional template**	Unattended items in WM maintained synaptically so stable attractor is reactivated by partial information	(Lavie and Fockert, 2005; Woodman et al., 2007)
Memory contents lead to obligatory **capture of attention**	Partial information pertaining to items in silent WM is amplified, leading to persistent activity / focus of attention	(Olivers et al., 2006; Soto et al., 2008)
WM capacity correlates with **complexity of task set**	One WM object corresponds to one stimulus-response pairing	(Conway et al., 2003; Duncan, 2010)

PFC subserves both WM and task set maintenance	Conjunctive neurons can flexibly bind actions to stimulus features, or groups of features, together	(Barch et al., 1997; Duncan et al., 2000)
Neural decoding of **unattended items** in WM is weak	Unattended items encoded in synaptic traces	(Lewis-Peacock et al., 2012; Sprague et al., 2016)
TMS to feature areas, or indiscriminate sensory stimuli, can **re-activate** representations	Neurons reactivated by TMS pulse that are connected synaptically to conjunctive neurons (i.e. unattended WM items) are selectively amplified by reciprocal synapses	(Rose et al., 2016; Wolff et al., 2017)
**TMS** to posterior cortex disrupts benefit conferred by focus of attention	Electrical activity i.e. persistent activation is disrupted by electrical stimulus, but synapses are not	(Zokaei et al., 2014a)
**rTMS to PFC** disrupts WM by altering posterior activity	Reducing conjunctive unit excitability reduces stability of attentional attractors	(Zanto et al., 2011)
Attention operates by frontal **amplification** of posterior feature-selective neurons	The attractor basin formed by mutual conjunctive and feature synapses allows conjunctive units to control gain in feature-selective neurons.	(Desimone and Duncan, 1995; Merrikhi et al., 2017; Moore and Armstrong, 2003)
Primacy effect not robust	Depends on focus of attention disengaging from previous trial items during ITI	(Baddeley, 2000)
**Transposition** errors	New conjunction unit not always activated for new items	(Farrell and Lewandowsky, 2004)
Binding must be **serial**	Conjunctive neurons encode all simultaneously active features.	(McLean et al., 1983; Treisman and Gelade, 1980)
More items mean faster decay	Focus of attention involves sustained firing so more robust to decay, whereas unattended items susceptible.	(Pertzov et al., 2016)
Errors report succeeding items more than prior items	More recently encoded items have stronger synaptic weights, and more likely to intrude.	(Farrell and Lewandowsky, 2004)
**Probe can interfere** with recall	Irrelevant features in probe suppress reactivation of items	(Souza et al., 2016)
**Reaction times** inversely related to accuracy	Time taken for conjunctive unit to activate features depends on synaptic strength and competition from other units	(McElree and Dosher, 1989; Pearson et al., 2014)
**Encoding rate** slower when more items stored	Items encoded briefly have weaker synaptic traces, thus are more susceptible to interference from other items in memory	(Bays et al., 2011)

Table S2: Psychological concepts corresponding to the model

**Binding**	Simultaneous activation of two feature neurons causes a single conjunction neuron to become active and form bidirectional connections to those feature neurons.
**Focus of attention**	Active representation in feature-selective neurons, coupled with a conjunctive unit that is simultaneously active. The two types of neuron are mutually excitatory and generate persistent activity.
**Unfocused item in WM**	Bidirectional increases in synaptic weights between a combination of place-coded feature neurons, and one conjunction neuron
**Recall**	Associative re-activation of a pattern of activity in feature neurons.
**Task set**	One of the feature dimensions represents motor-plan neurons. During encoding, the simultaneous activation of a motor plan and a perceptual feature causes a conjunctive unit to associate the two. Reactivating the perceptual feature triggers the motor program.
**Top-down control**	Conjunction neurons effectively amplify feature neurons that were previously encoded as WM items.
**Forgetting from WM**	Activation of feature neurons by an external stimulus provides new input. This input competes to be represented by conjunction neurons. The conjunction neuron with the most-similar connections will be activated and re-wire to encode the stimulus. This interfering stimulus thus overwrites and displaces an item previously in WM.

Movie S1: **Timecourse of activity during working memory encoding and recall**. (A) top left panel shows the instantaneous rate of change in weights Δ, (B) below is shown the current synaptic weights. (C) top right illustrates the object currently encoded by the feature neurons. The drawn intensity of each possible stimulus is the product of the activity of the corresponding feature neurons. During encoding this corresponds to the objects presented to the model. (D) lower panels show the feature neuron activity as a heatmap, as a function of time, and the activities of the four conjunctive neurons as traces. Delay period activity generally corresponds to the final item presented. Errors occur when one conjunctive neuron is active for two objects, or when one conjunctive unit fails to win the competition.

Supplementary References

Allen, R.J., Baddeley, A.D., Hitch, G.J., 2006. Is the binding of visual features in working memory resource-demanding? J. Exp. Psychol. Gen. 135, 298.

Baddeley, A., 2000. Short-Term and Working Memory. Oxf. Handb. Mem. 77.

Baddeley, A., 1996. The fractionation of working memory. Proc. Natl. Acad. Sci. 93, 13468–13472.

Barch, D.M., Braver, T.S., Nystrom, L.E., Forman, S.D., Noll, D.C., Cohen, J.D., 1997. Dissociating working memory from task difficulty in human prefrontal cortex. Neuropsychologia 35, 1373–1380. https://doi.org/10.1016/S0028-3932(97)00072-9

Bays, P.M., Gorgoraptis, N., Wee, N., Marshall, L., Husain, M., 2011. Temporal dynamics of encoding, storage, and reallocation of visual working memory. J. Vis. 11, 6–6. https://doi.org/10.1167/11.10.6

Bays, P.M., Husain, M., 2008. Dynamic Shifts of Limited Working Memory Resources in Human Vision. Science 321, 851–854. https://doi.org/10.1126/science.1158023

Cogan, G.B., Iyer, A., Melloni, L., Thesen, T., Friedman, D., Doyle, W., Devinsky, O., Pesaran, B., 2017. Manipulating stored phonological input during verbal working memory. Nat. Neurosci. 20, 279–286. https://doi.org/10.1038/nn.4459

Conway, A.R.A., Kane, M.J., Engle, R.W., 2003. Working memory capacity and its relation to general intelligence. Trends Cogn. Sci. 7, 547–552. https://doi.org/10.1016/j.tics.2003.10.005

Duncan, J., 2010. The multiple-demand (MD) system of the primate brain: mental programs for intelligent behaviour. Trends Cogn. Sci. 14, 172–179. https://doi.org/10.1016/j.tics.2010.01.004

Duncan, J., Seitz, R.J., Kolodny, J., Bor, D., Herzog, H., Ahmed, A., Newell, F.N., Emslie, H., 2000. A Neural Basis for General Intelligence. Science 289, 457–460. https://doi.org/10.1126/science.289.5478.457

Farrell, S., Lewandowsky, S., 2004. Modelling transposition latencies: Constraints for theories of serial order memory. J. Mem. Lang. 51, 115–135. https://doi.org/10.1016/j.jml.2004.03.007

Fries, P., Reynolds, J.H., Rorie, A.E., Desimone, R., 2001. Modulation of Oscillatory Neuronal Synchronization by Selective Visual Attention. Science 291, 1560–1563. https://doi.org/10.1126/science.1055465

Gorgoraptis, N., Catalao, R.F.G., Bays, P.M., Husain, M., 2011. Dynamic Updating of Working Memory Resources for Visual Objects. J. Neurosci. 31, 8502–8511. https://doi.org/10.1523/JNEUROSCI.0208-11.2011

Gregoriou, G.G., Gotts, S.J., Zhou, H., Desimone, R., 2009. High-frequency, long-range coupling between prefrontal and visual cortex during attention. Science 324, 1207–1210. https://doi.org/10.1126/science.1171402

Harrison, S.A., Tong, F., 2009. Decoding reveals the contents of visual working memory in early visual areas. Nature 458, 632–635. https://doi.org/10.1038/nature07832

Heinen, K., Feredoes, E., Ruff, C.C., Driver, J., 2017. Functional connectivity between prefrontal and parietal cortex drives visuo-spatial attention shifts. Neuropsychologia 99, 81–91. https://doi.org/10.1016/j.neuropsychologia.2017.02.024

Konecky, R.O., Smith, M.A., Olson, C.R., 2017. Monkey Prefrontal Neurons during Sternberg Task Performance: Full Contents of Working Memory or Most Recent Item? J. Neurophysiol. jn.00541.2016. https://doi.org/10.1152/jn.00541.2016

Lara, A.H., Wallis, J.D., 2014. Executive control processes underlying multi-item working memory. Nat. Neurosci. 17, 876–883. https://doi.org/10.1038/nn.3702

Lavie, N., Fockert, J.D., 2005. The role of working memory in attentional capture. Psychon. Bull. Rev. 12, 669–674. https://doi.org/10.3758/BF03196756

Lewis-Peacock, J.A., Drysdale, A.T., Oberauer, K., Postle, B.R., 2012. Neural evidence for a distinction between short-term memory and the focus of attention. J. Cogn. Neurosci. 24, 61–79.

Luck, S.J., Vogel, E.K., 1997. The capacity of visual working memory for features and conjunctions. Nature 390, 279–280.

McElree, B., 2006. Accessing Recent Events, in: Motivation, B.-P. of L. and (Ed.), . Academic Press, pp. 155–200. https://doi.org/10.1016/S0079-7421(06)46005-9

McElree, B., Dosher, B.A., 1989. Serial position and set size in short-term memory: The time course of recognition. J. Exp. Psychol. Gen. 118, 346–373. https://doi.org/10.1037/0096-3445.118.4.346

McLean, J.P., Broadbent, D.E., Broadbent, M.H.P., 1983. Combining attributes in rapid serial visual presentation tasks. Q. J. Exp. Psychol. Sect. A 35, 171–186. https://doi.org/10.1080/14640748308402123

Myers, N.E., Stokes, M.G., Nobre, A.C., 2017. Prioritizing Information during Working Memory: Beyond Sustained Internal Attention. Trends Cogn. Sci. 21, 449–461. https://doi.org/10.1016/j.tics.2017.03.010

Olivers, C.N.L., Meijer, F., Theeuwes, J., 2006. Feature-based memory-driven attentional capture: Visual working memory content affects visual attention. J. Exp. Psychol. Hum. Percept. Perform. 32, 1243–1265. https://doi.org/10.1037/0096-1523.32.5.1243

Pearson, B., Raskevicius, J., Bays, P.M., Pertzov, Y., Husain, M., 2014. Working memory retrieval as a decision process. J. Vis. 14. https://doi.org/10.1167/14.2.2

Pertzov, Y., Manohar, S., Husain, M., 2016. Rapid Forgetting Results From Competition Over Time Between Items in Visual Working Memory. J. Exp. Psychol. Learn. Mem. Cogn.

Rademaker, R.L., Chunharas, C., Serences, J.T., 2018. Simultaneous representation of sensory and mnemonic information in human visual cortex. bioRxiv 339200. https://doi.org/10.1101/339200

Rose, N.S., LaRocque, J.J., Riggall, A.C., Gosseries, O., Starrett, M.J., Meyering, E.E., Postle, B.R., 2016. Reactivation of latent working memories with transcranial magnetic stimulation. Science 354, 1136–1139. https://doi.org/10.1126/science.aah7011

Rowe, J.B., Toni, I., Josephs, O., Frackowiak, R.S.J., Passingham, R.E., 2000. The Prefrontal Cortex: Response Selection or Maintenance Within Working Memory? Science 288, 1656–1660. https://doi.org/10.1126/science.288.5471.1656

Sakai, K., Rowe, J.B., Passingham, R.E., 2002. Active maintenance in prefrontal area 46 creates distractor-resistant memory. Nat. Neurosci. 5, 479–484.

Sala, J.B., Courtney, S.M., 2007. Binding of What and Where During Working Memory Maintenance. Cortex 43, 5–21. https://doi.org/10.1016/S0010-9452(08)70442-8

Schvaneveldt, R.W., Chase, W.G., 1969. Sequential effects in choice reaction time. J. Exp. Psychol. 80, 1.

Scolari, M., Seidl-Rathkopf, K.N., Kastner, S., 2015. Functions of the human frontoparietal attention network: Evidence from neuroimaging. Curr. Opin. Behav. Sci., Cognitive control 1, 32–39. https://doi.org/10.1016/j.cobeha.2014.08.003

Smyth, M.M., Scholey, K.A., 1996. The relationship between articulation time and memory performance in verbal and visuospatial tasks. Br. J. Psychol. 87, 179–191. https://doi.org/10.1111/j.2044-8295.1996.tb02584.x

Soto, D., Hodsoll, J., Rotshtein, P., Humphreys, G.W., 2008. Automatic guidance of attention from working memory. Trends Cogn. Sci. 12, 342–348. https://doi.org/10.1016/j.tics.2008.05.007

Souza, A.S., Rerko, L., Oberauer, K., 2016. Getting More From Visual Working Memory: Retro-Cues Enhance Retrieval and Protect From Visual Interference. J. Exp. Psychol. Hum. Percept. Perform. https://doi.org/10.1037/xhp0000192

Sprague, T.C., Ester, E.F., Serences, J.T., 2016. Restoring Latent Visual Working Memory Representations in Human Cortex. Neuron 91, 694–707. https://doi.org/10.1016/j.neuron.2016.07.006

Stanton, P.K., Sejnowski, T.J., 1989. Associative long-term depression in the hippocampus induced by hebbian covariance. Nature 339, 215–218. https://doi.org/10.1038/339215a0

Szczepanski, S.M., Pinsk, M.A., Douglas, M.M., Kastner, S., Saalmann, Y.B., 2013. Functional and structural architecture of the human dorsal frontoparietal attention network. Proc. Natl. Acad. Sci. 110, 15806–15811. https://doi.org/10.1073/pnas.1313903110

Treisman, A.M., Gelade, G., 1980. A feature-integration theory of attention. Cognit. Psychol. 12, 97–136. https://doi.org/10.1016/0010-0285(80)90005-5

van Loon, A.M., Olmos-Solis, K., Fahrenfort, J.J., Olivers, C.N.L., 2018. Current and future goals are represented in opposite patterns in object-selective cortex. eLife 7. https://doi.org/10.7554/eLife.38677

Wolff, M.J., Jochim, J., Akyürek, E.G., Stokes, M.G., 2017. Dynamic hidden states underlying working-memory-guided behavior. Nat. Neurosci. 20, 864–871. https://doi.org/10.1038/nn.4546

Woodman, G.F., Luck, S.J., Schall, J.D., 2007. The Role of Working Memory Representations in the Control of Attention. Cereb. Cortex 17, i118–i124. https://doi.org/10.1093/cercor/bhm065

Yu, Q., Postle, B.R., 2018. Different states of priority recruit different neural codes in visual working memory. bioRxiv 334920. https://doi.org/10.1101/334920

Zanto, T.P., Rubens, M.T., Thangavel, A., Gazzaley, A., 2011. Causal role of the prefrontal cortex in top-down modulation of visual processing and working memory. Nat. Neurosci. 14, 656–661. https://doi.org/10.1038/nn.2773

Zhang, W., Luck, S.J., 2008. Discrete fixed-resolution representations in visual working memory. Nature 453, 233–235. https://doi.org/10.1038/nature06860

Zokaei, N., Manohar, S., Husain, M., Feredoes, E., 2014a. Causal evidence for a privileged working memory state in early visual cortex. J. Neurosci. 34, 158–162.

Zokaei, N., Ning, S., Manohar, S., Feredoes, E., Husain, M., 2014b. Flexibility of representational states in working memory. Front. Hum. Neurosci. 8, 853.
